# Advancement in the Treatment of Osteoporosis and the Effects on Bone Healing

**DOI:** 10.3390/jcm11247477

**Published:** 2022-12-16

**Authors:** Yevgeniya Kushchayeva, Iryna Pestun, Sergiy Kushchayev, Nataliia Radzikhovska, E. Michael Lewiecki

**Affiliations:** 1 Diabetes and Endocrinology Center, University of South Florida, Tampa, FL 33612, USA; 2 USF Health Informatics Institute, Tampa, FL 33612, USA; 3 Diagnostic Imaging and Interventional Radiology, Moffitt Cancer Center, Tampa, FL 33612, USA; 4 Clinical Hospital #15, Podil District of Kyiv, 04070 Kyiv, Ukraine; 5 New Mexico Clinical Research & Osteoporosis Center, Albuquerque, NM 87106, USA

**Keywords:** osteoporosis, bone-related surgeries

## Abstract

Osteoporosis (OP) is a major global health concern, with aging being one of the most important risk factors. Osteoarthritis (OA) is also an age-related disorder. Patients with OP and/or OA may be treated surgically for fractures or when their quality of life is impaired. Poor bone quality due to OP can seriously complicate the stability of a bone fixation construct and/or surgical fracture treatment. This review summarizes the current knowledge on the pathophysiology of normal and osteoporotic bone healing, the effect of a bone fracture on bone turnover markers, the diagnosis of a low bone mineral density (BMD) before surgical intervention, and the effect of available anti-osteoporosis treatment. Interventions that improve bone health may enhance the probability of favorable surgical outcomes. Fracture healing and the treatment of atypical femoral fractures are also discussed.

## 1. Introduction

Osteoporosis (OP) is a major public health concern that affects approximately 200 million people globally. The clinical consequences of this systemic disorder are an increased risk of fractures with an increased fracture severity [1]. Poor bone quality due to OP seriously complicates the surgical treatment of these fractures and stability of the bone fixation construct. Patients with OP having spine surgery are at an increased risk of pedicle screw loosening, instrumentation failure, pseudoarthrosis, vertebral fractures (VFs), proximal junctional kyphosis, and revision surgery [2]. Since OP is a disorder of aging and both aging and OP may affect the normal bone healing process, it is difficult to separate their negative effects on bone tissue [1].

## 2. Fracture Healing in Healthy Bone

**Figure 1:**
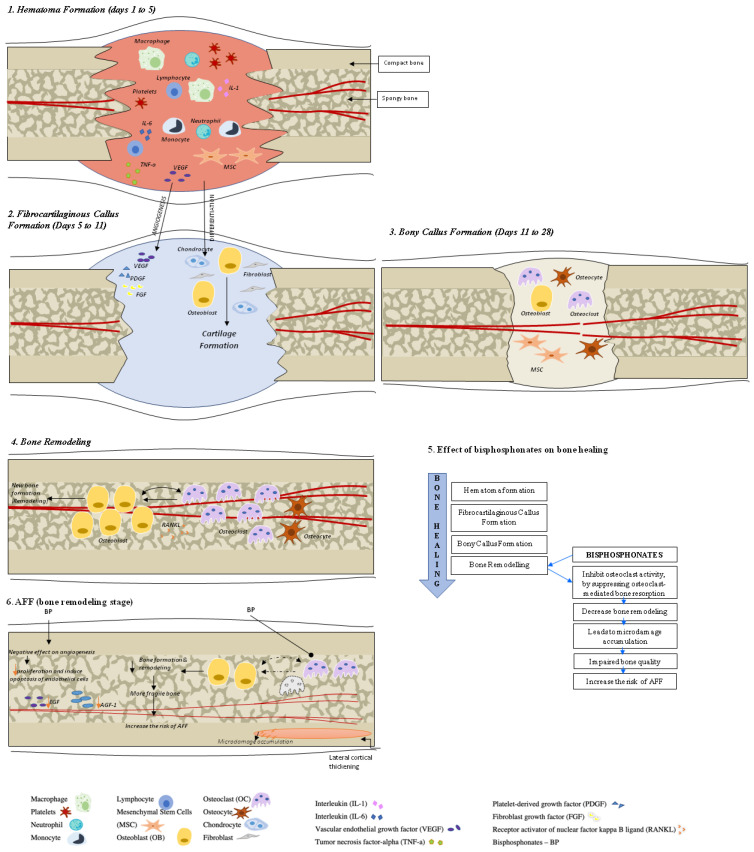
**Bone healing in healthy bone and after atypical femoral fracture (AFF).** There are four stages that describe the bone healing process: (1) hematoma formation with inflammation, (2) fibrocartilaginous callus formation, (3) bony callus formation, and (4) bone remodeling. During the first stage, a formed hematoma is composed of bone marrow and peripheral and intramedullary blood cells. The inflammatory cells (macrophages, neutrophils, lymphocytes, monocytes) and degranulating platelets infiltrate the hematoma between the fracture ends, causing acute inflammation and releasing cytokines and growth factors to stimulate the fracture healing process. During the fibrocartilaginous callus formation (stage 2), the soft callus is developed. The soft callus is a semi-rigid tissue able to provide mechanical support to the fracture and act as a template for the bony callus. The cartilaginous matrix is produced until the whole fibrinous/granulation tissue is replaced by cartilage. Angiogenic factors amplify the process of fracture healing vascularization. Further progress of bone regeneration occurs with the replacement of the primary soft cartilaginous callus with a hard bony callus during stage 3 (bony callus formation stage). The last stage represents bone remodeling that is characterized by high levels of bone resorption and formation markers and the migration of osteoblasts and osteoclasts with the hard callus that undergoes repeated remodeling. During this process, the central part of the callus is finally replaced by compact bone, whereas the callus edges are replaced by lamellar bone. See the test for more details.

Fracture healing is a complex, multistage, coordinated process commencing autonomously in the bone fracture area [3]. There are two principal histological types of bone healing: primary and secondary healing. Primary healing is rare and is based on the attempt of the cortical bone cells to re-establish the disrupted continuity directly, therefore requiring absolute stability and contact of the fragments, as may occur with a stress fracture and fractures treated with open reduction and internal fixation (ORIF) with plates and screws [4]. In contrast, secondary bone healing takes place in the majority of bony injuries and involves both intramembranous and endochondral ossification with the activation of committed osteoprogenitor cells of the periosteum and undifferentiated multipotent mesenchymal stem cells (MSCs). This type of bone healing involves callus formation [4]. Four stages typically describe the bone healing process: hematoma formation with inflammation, fibrocartilaginous callus formation, bony callus formation, and bone remodeling [5] (Figure 1).

***Hematoma Formation (Days 1 to 7)***. A hematoma is formed immediately after the fracture; it is composed of peripheral and intramedullary blood cells, and bone marrow cells. The inflammatory response, necessary for healing to progress, peaks within 24 h and is completed in 7 days. Hematoma coagulation within the medulla and between/around the fracture ends sets up a template for callus formation [6]. Inflammatory cells (macrophages, neutrophils, lymphocytes, monocytes) and degranulating platelets infiltrate the hematoma between the fracture ends, causing acute inflammation and releasing cytokines and growth factors that stimulate fracture healing [3,5,7].

***Fibrocartilaginous Callus Formation (Days 5 to 11)***. Chondrocytes and fibroblasts dominate on a cellular level at this stage; however, specific proportions of different cell types can vary among fractures. The soft callus produced by these cells is a semi-rigid tissue that is able to provide mechanical support to the fracture. At the same time, the soft callus acts as a template for the bony callus that will supersede it. The cartilaginous matrix is synthesized by proliferating chondrocytes derived from mesenchymal progenitors. This process lasts until the whole fibrinous/granulation tissue is replaced by cartilage [7].

Angiogenic factors, such as fibroblast growth factor (FGF), platelet-derived growth factor (PDGF), and vascular endothelial growth factor (VEGF) amplify the process of the fracture healing vascularization [3,8,9,10].

***Bony Callus Formation (Days 11 to 28).*** Further progress of bone regeneration occurs with the replacement of primary soft cartilaginous callus with a hard bony callus [6]. When endochondral ossification of the cartilaginous callus begins, the receptor activator of nuclear factor kappa B ligand (RANKL) is expressed. This stimulates the further differentiation of chondroblasts, osteoblasts, and osteoclasts, resulting in the resorption and calcification of the cartilaginous callus. At the same time, woven bone is laid down subperiosteally. The proliferation of newly formed blood vessels continues, allowing for the further migration of mesenchymal stem cells. At the end of this stage, a hard, calcified callus of immature bone is formed [5].

Fracture callus chondrocytes become hypertrophic in the course of their proliferation, while the extracellular matrix becomes calcified. This sequence of events is primarily controlled by macrophage colony-stimulating factor (M-CSF), RANKL, and osteoprotegerin (OPG), while the resorption of this mineralized cartilage is initiated by TNF-α [6,8]. It is also likely that, during this process, M-CSF, RANKL, and OPG help to recruit bone cells and osteoclasts to form woven bone [6].

***Bone Remodeling (lasting from months to years).*** The remodeling of bone tissue is the final stage of bone repair, characterized by high levels of bone resorption and bone formation markers [11]. Osteoblasts and osteoclasts continue migrating, and the hard callus undergoes repeated “coupled remodeling”, a process involving a balance of osteoclastic bone resorption and osteoblastic bone formation until the bone returns to its original state [5].

During the remodeling process, immature woven bone and underlying cartilage matrix are resorbed by osteoclasts and replaced with the lamellar bone. The fate of osteoblasts after completing bone formation is to undergo apoptosis, become bone lining cells, or embed themselves into the bone matrix as osteocytes. Cellular functions of both osteoclasts and osteoblasts are regulated by cytokines, which include RANKL and OPG [3].

## 3. Fracture Healing in Osteoporotic Bone

Osteoporotic bones are characterized by a low bone mineral density (BMD) and the degradation of the bone structure due to an imbalance in bone remodeling, with osteoclastic bone resorption exceeding osteoblastic bone formation [12]. Trabecular plates become rod-like, with thinning and perforations, resulting in bones that are weaker and more likely to break than normal bones [13].

The fragility of osteoporotic bone is associated with abnormal skeletal properties, which include a reduction in the mineral and protein contents that provide strength and stiffness to bone; a decrease in its ability to adapt to repetitive loads (fatigue resistance) to oppose to deformations (rigidity) and to absorb energy (resistance); and an increase in microdamage due to repetitive micro-stress [14].

The fracture healing of osteoporotic bone proceeds through the same phases as normal bone but the healing process may be prolonged [14] and complicated by a reduction in the number of mesenchymal cells and angiogenesis [1,12]. Comorbidities that can contribute to an impaired fracture healing in patients with osteoporosis include an advanced age, endocrine disorders, malignancies, hypogonadism, and medications (e.g., glucocorticoids, aromatase inhibitors) [1]. In the course of aging, there is an accumulation of micro-damage of bone tissue due to diminished physiological mechanisms of repair associated with a decreased osteoblast activity and an age-related decrease in bone marrow (BM) [12]. Moreover, in elderly people, osteoblasts have a reduced capacity to synthesize alkaline phosphatase, osteocalcin, collagen, and RUNX2, a transcription factor that induces the differentiation of multipotent mesenchymal cells into immature osteoblasts [15]; this contributes to a reduction in osteoprogenitor cells and the number and activity of osteoblasts, and a reduction in physiological stimuli as mechanical stress [14].

Sarcopenia and bone loss are age-related processes, each associated with a low BMD and osteoporosis [16]. It has been suggested that muscle contractions are the primary source of the mechanical load on bone tissue. Bone and muscle tissues interact via biomechanical and biochemical cross-talk. Both muscle and bone have been shown to secrete different regulatory factors into the systemic circulation. Some myokines produced by muscle tissue, such as myostatin, irisin, insulin-like growth factor 1 (IGF1), some interleukins, decorin, and osteoglycin, have effects on bone tissue. Osteokines produced by bone tissue, such as osteocalcin, prostaglandin E2, and Wingless-related integration site 3a (Wnt-3a), can target muscle tissue [17]. Secretory factors produced by muscle tissue may vary depending on the muscle activity, aging, and disuse [18]. Senescence-associated factors released by aging cells are harmful for musculoskeletal health. Senescent cells produce senescence-associated secretory phenotype (SASP), as demonstrated in an age-related OP in vivo model [19]. SASP released from bone marrow mesenchymal stem cells and osteoblasts inhibit bone formation [19,20].

Bone-muscle crosstalk is important for bone healing, with disruptions resulting in an altered biomechanical or biochemical interaction. Fracture healing is impaired in the case of muscle damage or atrophy [20].

## 4. Bone Turnover Markers (BTMs) in Fracture Healing (Table 1 and Table 2)

BTMs are biomarkers that are released into the systemic circulation during bone remodeling and can be measured in blood and urine. They are classified according to whether they primarily represent bone resorption or bone formation [21]. Markers of bone formation include serum osteocalcin (OC), bone-specific alkaline phosphatase (BALP), and the N-terminal propeptide of type I collagen (P1NP); the most sensitive markers of bone resorption are crosslinked C- (CTX) and N- (NTX) telopeptides of type I collagen [11,22,23]. The use of serum P1NP and CTX has been recommended by the International Osteoporosis Foundation (IOF), the International Federation of Clinical Chemistry (IFCC) Bone Marker Standards Working Group, and the National Osteoporosis Foundation (NOF) as bone turnover reference markers for fracture risk prediction and for OP treatment evaluating and monitoring [24,25].

As BTMs do not change significantly in the first few hours after fracture, immediate post-fracture samples may provide information on the baseline state of the bone turnover [26]. BTMs will significantly increase within the first weeks after a fracture. BTMs may be correlated with the fracture size and healing time (Table 1). Extensive fractures need more time to move through the repair cycle than small fractures and are characterized by the release of a large amount of BTMs. Moreover, the duration of BTM elevation depends on the extensiveness of the fracture, with minor fractures such as the forearm being elevated for 6 months and up to a year after more extensive fracture, such as a hip fracture [27,28].

The interpretation of BTM levels is confounded by pre-analytical and analytical variability. There is also a relatively low bone specificity, since collagen metabolism is not limited to bone [11]. Drugs (e.g., glucocorticoids, anticonvulsants), menopausal status, age, gender, pregnancy/lactation, renal insufficiency, and immobility may influence BTM levels [29,30] (Table 2).

**Table 1:** Bone turnover markers, their activity in fracture healing, and limitations of BTMs use.

Bone Turnover Marker	Origin	Expected Change in Level during the Fracture Healing	Conditions That Affect BTM Levels
P1NP	Product of the type I procollagen degradation during the procollagen-to-collagen conversion. Cells—osteoblasts [23]	Peak at 12 weeks after fracture, remains elevated at 24 weeks [11,31].	Antiresorptive treatment (such as estrogen and BPs) lowers procollagen peptide levels [32]. Anabolic agents such as TPTD and Rmab increase procollagen peptide concentrations [32,33,34]. Renal function deviations have no influence on P1NP, so this marker can be used in patients with CKD [21].
BALP	Enzyme needed in osteoid formation and mineralization [35]. Cells—osteoblasts.	The level is elevated at 4 weeks after fracture of the tibial shaft and remains increased at 1 year [11].	BALP has several advantageous features, which include low circadian variation due to its half-life of 1 to 2 days, stability of samples, broad availability of assays, and lack of renal clearance [36]. BALP can be used in patients with CKD [37].
OC	Non-collagen protein is a kind of calcium-binding protein, vitamin-K-dependent, associated with bone mineralization [23]. Cells—osteoblasts.	The level is elevated at 24 weeks after fracture of the tibial shaft and at 1 week after distal radial fracture [11].	OC is metabolized in liver and kidneys and is influenced by renal clearance, with higher levels of OC that occur in CKD. Some anticoagulants (such as a high dose of heparin for one week) can reduce OC level by 40% [32,36]. OC is affected by renal clearance and has circadian rhythm with peak at around 4 AM [36].
CTX	Degradation of mature type I collagen marker. CTX is formed in the process of bone resorption mediated by cathepsin-K [38].	The level rises in the first week after fracture, with peak at 4 weeks after fracture, and remains elevated throughout fracture healing [11].	Fasting morning samples are important for optimal clinical use since fasting reduces circadian variations [32]. This biomarker decreases rapidly in the course of antiresorptive therapy [38].
TRACP5b	The serum enzyme activity reflects the number of active osteoclasts [35]. Cells—resorbing osteoclasts	Peak is approximately seven days after osteosynthesis and after two weeks in fractures, remaining high at 24 weeks [11].	As this marker is not secreted in urine, it can be used in CKD patients [29]. TRAP5b levels are not affected by food intake as well, but they feature diurnal variation and increase immediately after exercise. In addition, TRAP5b samples are unstable at room temperature [38].

BALP—bone-specific alkaline phosphatase; BPs—bisphosphonates; BTMs—bone turnover markers; CKD—chronic kidney disease; CTX—crosslinked C telopeptide of type 1 collagen; OC—osteocalcin; P1NP—N-terminal propeptide of type I collagen; Rmab—romosozumab; TPTD—teriparatide; TRACP5b—tartrate-resistant acid phosphatase 5b.

**Table 2:** Factors affecting bone turnover marker levels.

Factor	Effect on BTM Levels
Age and gender	Highest levels are in infancy and remain high in childhood, with a nadir in women in the fourth decade and the fifth decade in men. Men <35 years old have higher BTMs vs. women due to longer lasting bone consolidation into young adulthood in men [38,39].
Menopausal status	Bone formation and resorption markers are higher during a few months following the menopause onset and both of these levels remain elevated thereafter [32,36].
Fractures	Elevation of bone resorption markers levels occurs within the first four weeks after a fracture, followed by increase in bone formation markers. Elevation of BTMs levels is estimated as 20–50% and may persist for up to six months [32].
Pregnancy and lactation	BTMs are increasing in the course of pregnancy. They reach higher values in the third trimester and even higher levels occur postpartum [32]. Elevation of levels of both formation (BALP and P1NP) and resorption markers (cross-links and telopeptides) start from the second trimester of pregnancy. These levels reach significantly higher values than before pregnancy [32]. The serum OC concentration decreases in the first two trimesters, with normalization in the third trimester and after delivery. Lowering of bone markers levels occurs postpartum over a period of 6–12 months, with slower decline during the lactation period [32,36].
Drugs intake	Glucocorticoid therapy reduces serum of formation markers (OC and P1NP by up to 40% to 50%) within a few days of therapy initiation, with little effect on bone resorption markers [36]. Intake of anticonvulsants may result in elevation of BTMs levels. It is essential to pay close attention to intake of corticosteroids, anticonvulsants, heparin, and GnRH agonists [36].
Fasting status/food intake	Feeding causes suppression of BTMs, with more pronounced effect on resorption markers, which can be decreased by 20–40% in contrast to bone formation markers (10% suppression). CTX level decreases by 20% after breakfast) [29,36].
Bed Rest/Immobility	2–4 days of bed rest leads to a significant bone resorption markers elevation and, after 1 week, these levels increase by 30% to 50% vs. bone formation markers, which remain unchanged or increase only slightly [36].

BALP—bone-specific alkaline phosphatase; BTMs—bone turnover markers; GnRH—gonadotropin-releasing hormone; OC—osteocalcin; P1NP—N-terminal propeptide of type I collagen; CTX—crosslinked C telopeptide of type 1 collagen.

## 5. Assessment of Bone Health before Bone Surgery

Poor bone health in patients undergoing bone surgery is a major risk factor for fixation failure, since the ability of screws to resist pullout from bone is directly related to BMD and bone quality [40,41]. Osteoporotic bone is less dense, with a thinning of trabeculae, poor vascularity, and disruption of the bone remodeling [41].

The prevalence of osteopenia (T-score between −1.0 and −2.5), OP (T-score ≤−2.5), and fractures increases with an advancing age. In a study of 1,321 patients having spine surgery, OP was diagnosed in 14.5% of male and 51.3% of female patients older than 50 years, and osteopenia was diagnosed in 46.1% of male and 41.4% of female patients older than 50 years [42]. Moreover, older patients are more likely to experience surgical complications [43]. Of note, low lumbar spine (LS) BMD is correlated with a periprosthetic bone loss after total hip arthroplasty [44]. Postmenopausal women with a low BMD have been shown to have a higher subsidence of the femoral stem in the cementless total hip arthroplasty [45]. A bone loss of approximately 30–40% is needed before it can be detected with plain X-rays [46]. In a study of patients with radiographic osteopenia who were tested by DXA, approximately 1% were diagnosed as having a normal BMD, 49% osteopenia, and 38% osteoporosis [47]. Of those with a radiographic diagnosis of osteoporosis, approximately 13% had a normal BMD by DXA, whereas 45% had osteopenia and 42% osteoporosis [47].

Based on the Best Practice Guidelines for Assessment and Management of Osteoporosis in Adult Patients Undergoing Elective Spinal Reconstruction (2022), screening for OP is recommended in all patients over an age of 65 years. Based on the expert panel, for 50–64-year-old patients, BMD testing should be performed when at least one of the following risk factors is present: chronic glucocorticoid use; history of cancer treatment known to affect the BMD; history of metabolic bone disease or fragility spine or hip fracture; uncontrolled diabetes mellitus (>10 years of poor control); chronic kidney disease (CKD), defined as glomerular filtration rate (GFR) <60 mL/min per 1.73 m${}^{2}$); a high fracture risk based on the fracture risk algorithm, FRAX, without known BMD; vitamin D deficiency; patients who are current smokers, alcohol users, or have a limited mobility; and patients with a history of failed spine surgery. Patients under 50 years of age should be tested in case of previous fragility fracture, chronic glucocorticoid use, metabolic bone disease, cancer treatment, or chronic kidney disease [2].

Patients with CKD have a higher fracture risk compared to the general population; this increases in a graded manner with the worsening of the GFR [48]. The risk of fracture was shown to be approximately 1.5 and 3 times higher among women older than 65 years and GFR 45–59 or <15, respectively, vs. patients with a normal GFR (>60 mL/min per 1.73 m${}^{2}$) [48]. The risk of fractures has been reported to be 16% higher in patients with end-stage renal disease (ESRD) vs. pre-dialysis CKD patients, especially in the hip [49]. Long-term dialysis and CKD mineral and bone disorder (CKD-MBD) increase the risk of osteonecrosis and joint arthropathy requiring total joint arthroplasty (TJA) [50]. Patients with ESRD have inferior outcomes after TJA, including periprosthetic joint infections, surgical site complications, and mortality [51]. Hemodialysis patients has been shown to have 16% of the revision rate after total hip arthroplasty (THA) [52].

In patients with CKD stage 5D, dual-energy X-ray absorptiometry (DXA) BMD measurement has been shown to be useful for predicting fractures for women with low serum parathyroid hormone (PTH) levels. In contrast, BMD measurement had discriminatory power for prevalent spine fracture regardless of gender or PTH [53]. Based on the most recent Kidney Disease: Improving Global Outcomes (KDIGO) 2017 clinical guidelines, in patients with CKD G3a-G5D with evidence of CKD-MBD and/or risk factors for OP, DXA evaluation was recommended [54]. Each standard deviation (SD) decrease in the total hip BMD was associated with a fracture odds ratio of 1.75 [55].

## 6. Imaging in Fracture Assessment (Table 3)

### 6.1. Dual-Energy X-ray Absorptiometry

DXA is the gold standard method for diagnosing osteoporosis before a fracture occurs and for assessing the fracture risk, with the ability to measure the BMD at the LS, hip, and radius. There are limitations of DXA. It should not be performed in pregnant women due to radiation exposure. Poor patient positioning or the presence of artifacts (e.g., surgical hardware, laminectomy, vertebral augmentation, degenerative changes) may falsely increase or decrease the BMD [56,57,58,59,60]. The hip BMD can be altered by improper hip positioning, extrinsic artifacts, avascular necrosis, metastases, and primary bone lesions.

Trabecular bone score (TBS). The TBS is a textural index that provides an indirect measurement of the bone microarchitecture based on a gray-level pixel variation in the LS DXA image [61]. It predicts the fracture risk independently of BMD. It is a validated input for the fracture risk algorithm, FRAX. It is potentially useful for monitoring the skeletal effects of anabolic therapy (i.e., teriparatide (TPTD), abaloparatide, romosozumab (Rmab), and possibly denosumab (Dmab), but is less likely to show changes in BMD with bisphosphonate (BP) therapy [62]. A low TBS is associated with a higher risk of osteoporotic fractures in postmenopausal women and is lower in patients with a prior osteoporotic fracture, regardless of whether the T-score is in the osteoporotic or osteopenic range [63,64,65,66]. Each SD decline in the TBS confers a 35% greater age-adjusted risk of any major osteoporotic fractures [67].

**Table 3:** Benefits and limitations of imaging modalities for bone health assessment.

Imaging Modality and What Can Be Measured	Benefits	Limitations
**DXA** BMD Z-score T-scores TBS	Current gold standard for BMD assessment Indirect measurement of bone microarchitecture with TBS Validated and readily available Short examination time Low radiation exposure (approximately 0.001 mSv) Low cost Used for diagnosis of osteoporosis, assessment of fracture risk, and monitoring BMD changes with or without treatment	Cannot be used: − In pregnant women due to radiation exposure; − When patient weight exceeds weight limit of DXA table, and at invalid skeletal sites (e.g., surgical hardware, severe skeletal deformities) Skilled technologist required Does not distinguish between cortical and trabecular bone BMD measurement influenced by bone size and aortic-vascular calcification
**REMS** DXA-equivalent BMD, Z-score, T-score Fragility score	Non-ionizing radiation Can be used in case of instrumentation presence Calcifications or osteophytes are excluded due to the identification of unexpected spectral features Not position-dependent in case of bone deformities, immobilization	Does not distinguish between cortical and trabecular bone Skilled technologist required Not widely available New technology with limited experience in clinical practice
**Plain X-ray** Structural bone changes	Diagnosis of fracture(s) and skeletal deformities Recognition of severe demineralization (radiographic osteopenia) Moderate cost Readily available	>30% of bone loss is needed before demineralization is visible on X-ray Radiation exposure much higher than DXA: chest X-ray ∼0.06–0.25 mSv; lumbar spine X-ray ∼1.5 mSv Cannot be used for quantification of BMD
**QCT, pQCT**	Measures volumetric BMD in cortical and trabecular compartments for hip and spine (QCT) and peripheral skeletal sites (pQCT) DXA-equivalent hip T-scores can be generated	Expensive High radiation Limited availability
**HR-pQCT** Structural bone changes, mechanical properties, microarchitecture	Measures volumetric BMD and bone microarchitecture at the distal tibia and radius	Expensive Very limited availability Primarily a research tool Radiation exposure more than DXA or plain X-ray
**Opportunistic CT** Structural changes, L1 HU	Can diagnosis fractures and calculate BMD with CT imaging obtained for non-skeletal indications Minimal additional cost since image is already available	High radiation exposure (∼8 mSv for routine chest CT and ∼15 mSv for CT abdomen and pelvis).
**Opportunistic MRI** Structural changes, microarchitecture Acute vs. chronic bone changes M- score	No radiation exposure Can diagnose fractures and assess recency of fracture with MRI obtained for non-skeletal indications Minimal additional cost since image is already available	Not validated for BMD assessment Lower spatial resolution than CT

BMD—bone mineral density; CT—computed tomography; DXA—dual-energy X-ray absorptiometry; FRAX—fracture risk assessment; HR-pQCT—high-resolution peripheral quantitative computed tomography; HU—Hounsfield unit; MRI—magnetic resonance imaging; mSv—millisievert; pQCT—peripheral quantitative computed tomography; QCT—quantitative computed tomography; REMS—radiofrequency echographic multispectrometry; TBS—trabecular bone score.

### 6.2. Radiofrequency Echographic Multi-Spectrometry (REMS)

This is an ultrasound technology that generates T-score values for the LS and hip that are correlated with DXA T-scores; it was cleared for clinical use by the US Food and Drug Administration (FDA) in October 2018. REMS performs an analysis of bone with non-ionizing radiation using ultrasound signal backscattering [68,69,70]. The BMD is calculated through comparisons of the patient’s specific bone spectrum with a reference database of ultrasound spectral models, with the generation of corresponding T-score and Z-score values derived from a normative reference database (National Health and Nutrition Examination Survey, NHANES) [68]. This approach has been validated through several national studies [69,70,71,72,73].

The precision and diagnostic accuracy of REMS in comparison with DXA have also been validated [68,71]. A high linear correlation has been found in LS and hip BMD measured by standard DXA and by REMS. The results demonstrate that REMS has a high accuracy for OP diagnosis, with a sensitivity and specificity over 90% and a diagnostic concordance of approximately 86% for the spine and hip. The REMS performance has been shown to have a sensitivity and specificity for the identification of patients with OP of over 90% for the positive predictive value (PPV, in the range of 82–86%) and, for the negative predictive value (NPV), a sensitivity and specificity of over 97% for spine and hip sites [74]. REMS is radiation-free; its use has recently been demonstrated in women during pregnancy [75]. Its use requires a trained and skilled operator.

### 6.3. Opportunistic Computed Tomography (CT)

L1 vertebral body trabecular attenuation expressed as Hounsfield units (HU) by CT is an alternative and reliable method used to determine BMD [76]. L1 trabecular attenuation has been proposed as a method to identify individuals at a high risk for fracture [76]. The L1 vertebral level is an optimal target for opportunistic screening because it is easily identifiable as the first non-rib-bearing vertebra. It is included on all abdominal and chest CT examinations and typically has few degenerative changes. It has the closest correlation with the BMD measured by DXA compared with other vertebral levels [76]. Based on recommendations by the International Society for Clinical Densitometry (ISCD), opportunistic CT-based attenuation using (HU) can be used to estimate the likelihood of osteoporosis (L1 HU < 100) and normal (L1 HU > 150) and support decisions regarding bone health assessment [77]. L1 HU thresholds of 99 and 136 HU for the diagnosis of OP have also been proposed [78,79,80].

Patients undergoing elective spinal reconstruction should have a DXA assessment if the lumbar HU is less than or equal to 150. However, spine CT is not considered as equal to DXA for the diagnosis of osteoporosis [2].

In patients who are not eligible for DXA due to the presence of instrumentation or scoliosis with significant spine deformity and who have a CT performed for any other reasons with a visible L1 vertebral body, an “opportunistic” measurement of HU can be performed. A CT scan can also identify previously unrecognized VFs. CT is generally not useful for monitoring patients due to the high radiation exposure.

### 6.4. Opportunistic Magnetic Resonance Imaging (MRI)

Because of the multiplanar imaging capability and ability to discriminate different types of tissue, MRI is sensitive in detecting the presence of stress fractures, avulsions, or hidden fractures, especially in settings of trauma [81]. Trabecular injuries that lead to hemorrhage, edema, or hyperemia can be seen in areas of poorly marginated signal intensity alteration in the cancellous bone and bone marrow on MRI [81]. The bone marrow signal on T1-weighted MRI images has been shown to negatively correlate with BMD and OP [82,83].

Since there is an inverse relationship between BMD, fragility fractures, and adipose tissue in vertebral bone marrow, th M-score has been proposed as a new quantitative method for OP screening on lumbar-spine MRI [84,85,86,87]. The M-score is a measurement of the signal-to-noise ratio (SNR) in L1–L4 that negatively relates to the BMD. M-score thresholds have been suggested: <1.26 for a normal bone density, 1.26 to 2.05 for osteopenia, and >2.05 for OP [87].

### 6.5. Quantitative Computed Tomography (QCT)

QCT can be used for the measurement of volumetric BMD in cortical and trabecular compartments of the spine and proximal femur. However, when both DXA and QCT are available, DXA is the preferred method for BMD assessment [77]. QCT may be preferable over DXA for some patients, including those with an extremely high and low bone mass [88,89,90]. Recently, a better prediction of osteoporotic fractures has been shown for opportunistic QCT vs. DXA in neurosurgical and oncologic patients. Moreover, in 56% of patients with a new VF, the diagnosis of osteoporosis was missed with T-score classification by DXA, in contrast to opportunistic QCT, for which, the rate of missed osteoporosis diagnoses was 19% [91].

Based on recommendations of the ISCD (2019), femoral neck and total hip T-scores calculated from 2D projections of quantitative computed tomography (QCT) data (2D data can be obtained from 3D QCT) are equivalent to the corresponding DXA T-scores for the diagnosis of OP in accordance with the WHO criteria [92,93]. There is no consensus on the diagnostic standards based on spine QCT. The proposed standard American College of Radiology (ACR) QCT-cutoff values for QCT trabecular spine BMD are >120 mg/cm${}^{3}$ for normal bone, 80–120 mg/cm${}^{3}$ for osteopenia, and <80mg/cm${}^{3}$ for OP [91,94].

## 7. Anti-Osteoporosis Medications and Bone Health before Orthopedic Surgery

### 7.1. Vitamin D

Vitamin D is essential for calcium homeostasis and bone metabolism. Cholecalciferol is hydroxylated in the liver to calcidiol (calcifediol) [25(OH)${}_{2}$D${}_{3}$]), the major circulating form of vitamin D, which is hydroxylated again in the kidneys to the active form, 1,25-dihydroxyvitamin D [1,25(OH)${}_{2}$D${}_{3}$] (calcitriol). Vitamin D is essential for the absorption of calcium and phosphate from the small intestine. Vitamin D sufficiency is usually assessed by measuring blood levels of 25(OH)${}_{2}$D${}_{3}$, with no consensus for optimal levels. A 25(OH)${}_{2}$D${}_{3}$ value ≤ 20 ng/mL may be considered as vitamin D deficiency, from 21–29 ng/mL as vitamin D insufficiency, and ≥ 30 ng/ mL as normal [95]. The measurement of 1,25(OH)${}_{2}$D${}_{3}$ is generally not recommended for assessing the vitamin D status, since its concentration fluctuates widely during the day and it has a much shorter half-life compared with 25(OH)${}_{2}$D${}_{3}$ (approximately 15 h vs. 15 days, respectively). The serum concentration of 1,25(OH)${}_{2}$D${}_{3}$ is 1000-fold lower than that of 25(OH)${}_{2}$D${}_{3}$[96]. Moreover, even with vitamin D deficiency, a compensatory elevation of PTH levels leads to an increase in kidney production of 1,25(OH)${}_{2}$D${}_{3}$ so that levels in circulation may be maintained within the normal range. The measurement of 1,25(OH)${}_{2}$D${}_{3}$ should be considered in patients with CKD, rickets, or granulomatous diseases [95].

Active 1,25(OH)${}_{2}$D${}_{3}$ binds to vitamin D receptors (VDRs) in the kidneys, intestine, parathyroid glands, and bones. The 1,25(OH)${}_{2}$D${}_{3}$-VDR complex is critical for the normal coupling of bone remodeling [97]. An intact 1,25(OH)${}_{2}$D${}_{3}$-VDR system is important for both basal and PTH-induced osteoclastogenesis; 1,25(OH)${}_{2}$D${}_{3}$ administration inhibits PTH synthesis and parathyroid cell growth, thus rendering 1,25(OH)${}_{2}$D${}_{3}$ therapy effective in treating the secondary hyperparathyroidism of chronic kidney disease (CKD) [98]. Vitamin D deficiency can lead to secondary hyperparathyroidism and an increased bone resorption. An elevated PTH was found in 35.4% of patients before spinal surgery [99]. Among patients after spinal fusion, 84% were found to have vitamin D deficiency or insufficiency [100]. Extremely low serum 25(OH)${}_{2}$D${}_{3}$ levels (median value of 7.2 ng/mL) were found in elderly patients with hip fractures, with 71.1% of the patients having vitamin D levels below 12 ng/mL [101].

Evidence on the effect of vitamin D deficiency on bone healing in humans is limited. A reduction in hip fracture risk and increased hip BMD was demonstrated in postmenopausal women treated with calcium and vitamin D supplementation in the Women’s Health Initiative clinical trial [102]. A metanalysis of 29 clinical trials (*n* = 63,897) supported a risk reduction in fractures of all types and a reduced rate of bone loss at the hip and spine, with daily calcium doses of ≥1200 mg and vitamin D doses of ≥800 IU [103]. It has been speculated that the effect of vitamin D in preventing fractures may be due to its action on muscle tissue; low vitamin D has been associated with an increased risk of sarcopenia, reduced muscle strength, and a reduction in the ability to perform daily activities and falls [104,105,106,107,108,109].

The evidence suggests that vitamin D deficiency may be associated with an impaired postoperative neurologic function, diminished quality of life, and increased risk of pseudoarthrosis [110]. Based on a systemic review of outcomes after spinal fusion, patients presenting with vitamin D deficiency have lower fusion rates and higher rates of persistent low back pain postoperatively [100]. Vitamin D deficiency is an independent predictor of nonunion, with a significantly longer fusion time [110,111]. Postoperative vitamin D supplementation in deficient patients was reported to lead to significant improvements in low back pain intensity, patient-reported outcomes scores, and fusion rates [100].

### 7.2. Anti-Osteoporosis Medication

Orthopedic or neurosurgical teams provide the initial treatment for most patients with fractures. Since there is a high risk for re-fracture in patients during the first 2 years after the index fracture (imminent fracture risk), patients who suffered fragility fractures should be promptly treated for secondary fracture prevention. The overall risk of another fracture after an index fragility fracture has been described as 7.6% and 11.6% within the first 1 to 2 years, respectively [112]. The risk of second fracture is age-dependent and increases by 4% each year, with it being more common in women than men [112,113]. The risk of a fracture within 2 years is higher after an initial VF (16.5%) in comparison to other types of fractures, such as humerus/proximal humerus/shoulder fractures (13%) and hip fracture (12.8%) [112]. For secondary fracture prevention, fracture liaison services (FLSs) have been effective. FLS is a systematic program used for identifying patients with fractures, usually in the hospital setting, entering them into a registry and following them to assure that they are evaluated and appropriately treated. FLSs have been shown to increase the rate of BMD testing and treatment initiation, and reduce the risk of re-fracture and mortality [114]. Furthermore, the Project ECHO model™ (Extension for Community Healthcare Outcomes) with a focus on OP care was initially developed at the University of New Mexico (https://hsc.unm.edu/echo/partner-portal/programs/new-mexico/bone-health/, accessed on 1 December 2022) and is now a world-wide known recourse of an evidence-based interactive distance-learning provided by many institutions (Bone Health ECHO, Own the Bone ECHO, Rare Bone Disease TeleECHO, and many other ECHO projects) to help providers improve their knowledge and treat patients with osteoporosis.

Available antiosteoporosis medications can be divided into three groups: (1) antiresorptive medications (the inhibition of bone remodeling), (2) anabolic medications (the stimulation of bone modeling, with an increase in bone formation > resorption), and (3) dual-effect (resorption inhibition and the stimulation of formation).

Currently, there is no FDA approval for anti-osteoporosis treatment/prophylaxis prior to bone surgery to improve surgical outcomes. Medical therapy before planned spinal surgery is recommended for patients, with OP defined as T-score < −2.5 [2]. However, it is also reasonable to consider it for patients with poor bone health, especially those with prior adult fractures and a high fracture probability according to FRAX (10-year probability for major osteoporotic fracture ≥20% or 10-year probability for hip fracture ≥3%).

The antiresorptive group includes bisphosphonates (BPs) and denosumab (Dmab) (Table 4 and Table 5).

BPs are small molecular drugs that affect bone tissue with no affinity to other tissue. BPs have a highly selective effect on osteoclasts and suppress osteoclast-mediated bone resorption, slowing the bone remodeling cycle. However, since resorption and formation are coupled during the remodeling cycle, BPs inhibit both formation and resorption, with a more pronounced effect on resorption.

BPs are nonhydrolyzable synthetic analogs of inorganic pyrophosphate with a high affinity for hydroxyapatite crystals in bone tissue, and are preferentially incorporated into active bone remodeling sites, particularly resorption areas [128,129]. BPs reach bone by entering the bone extracellular space through paracellular transport. BPs then bind to free hydroxyapatite on the bone surface [130]. BP molecules that are incorporated into osteoclasts promote apoptosis. When released from osteoclasts, BPs can reattach to bone locally or be released into the systemic circulation and reattach elsewhere in the skeleton, resulting in a skeletal half-life of many years [131].

The BP mechanism of action is the inhibition of the protein synthesis that is required for osteoclast function, such as the maintenance of the cytoskeleton and ruffled border formation [132,133]. Morphological changes in BP-exposed osteoclasts are described as diminishing the ruffled border and disrupting the cytoskeleton [134,135]. Oral nitrogen-containing BPs have a very low rate of gastrointestinal (GI) absorption (<1%) [128,136], with an approximately 40–60% skeleton retention, and the rest of the drug being rapidly eliminated by the kidneys [128,130]. BPs are not metabolized to inactive products [135].

The highest skeletal concentration of BP is found in the spine due to its high content of metabolically active trabecular bone [137,138,139]. BPs do not improve the trabecular microarchitecture. The increase in BMD with BPs occurs due to the enhanced secondary mineralization of preformed osteons and closure of the existing skeletal remodeling space [140,141].

BPs decrease the risk of fractures by 40–70% in the spine and 20–50% in the hip, and decrease the risk of non-VFs by 15–39% [142]. Based on a metanalysis of 10 randomized clinical trials (23,382 postmenopausal women), approximately 1 year (12.4 months) of treatment is the minimal time for BPs to be beneficial for preventing one non-VF per 100 postmenopausal women, and, with 200 women, OP needs 20.3 months of BP therapy to prevent one hip fracture and 12.1 months to avoid one clinical VF [143]. This suggests that BPs are most likely to be effective in women, with a life expectancy of at least 1 to 2 years.

BPs differ by how tightly they bind and detach from bone, with oral risedronate (RIS) being less tightly bound and quicker to be released from bone than zoledronic acid (ZOL), an intravenous BP that is characterized by tight bounding and a slow release from bone tissue [135]. The BP skeletal uptake and bone retention depends on the potency of BP—the magnitude of the antiresorptive effect. The length of the suppression of bone turnover also depends on the potency of BP, with ZOL being the most potent BP [128].

Prolonged therapy with BPs, with up to 6 years of annual ZOL, up to 7 years of RIS, and up to 10 years of alendronate (ALN), has been shown to maintain bone density [144,145,146,147]. A fracture prevention benefit may last for at least 3 years after stopping ZOL, 2–3 years after stopping ALN, and 1–2 years after stopping ibandronate and RIS. Current recommendations suggest reassessing the indications to continue BP therapy after 3–5 years of treatment [148]. There is evidence that the discontinuation of BPs does not increase the fracture risk in 3 and 5 years based on the Fracture Intervention Trial Long-term Extension (FLEX) and Health Outcomes and Reduced Incidence with Zoledronic Acid Once Yearly-Pivotal Fracture Trial (HORIZON-PFT) studies [144,147]. However, there is a decrease in the BMD after the discontinuation of BPs [144,149,150]. Based on the FLEX trial, the mean loss in BMD after ALN was stopped was 3.4% at the total hip (TH), 1.4% at the femoral neck (FN), and 1.5% at the LS. RIS was shown to have a 34% greater risk of hip fracture with a drug holiday of longer than 2 years, in contrast to ALN, suggesting that drug holidays may need to be shorter for patients previously treated with RIS than with ALN [151]. A poor adherence to therapy with BPs, or poor GI absorption, may be recognized when BTMs are not suppressed as expected after a few months of therapy [152].

BPs have no adverse effects on bone healing in patients with spinal fusion, with no difference in screw loosening between BP and controls in a study of patients who underwent lumbar fusion [153]. The infusion of ZOL 3 days and 1 year after lumbar interbody fusion surgery was associated with an increased rate of solid fusion (75% vs. 56% in the control group), lower incidence of subsequent compression fractures (19% vs. 51%), pedicle screw loosening (18% vs. 45%), and cage subsidence >2 mm (28% vs. 54%) after 2 years of follow-up [154].

BPs have no effect on clinically detectable delays to indirect bone healing regardless of the timing of the BP delivery in relation to the fracture [155]. However, in long-term BP users (>5 years) BP-associated atypical femoral fracture (AFF) may rarely occur, with a delay in healing in 26% of cases [156]. Treatment with BP, such as ZOL, has been recommended as second-line therapy for enhancing spine surgery outcomes if anabolic medications cannot be used for any reason [2].

It has been documented that the sequence of a bone-forming agent followed by antiresorptive therapy has the potential to provide substantially larger BMD improvements than treatment with an antiresorptive agent first [157] (Table 4).

***Dmab*** is a fully human monoclonal antibody that binds to the receptor activator of nuclear factor-κB ligand ((RANKL), preventing it from binding to its receptor, RANK. RANKL is required for osteoclast precursor differentiation via interaction with RANK, which is expressed on many cell types, including osteoclast precursors and mature osteoclasts. Preventing RANKL-RANK interaction leads to the inhibition of osteoclast formation and function, leading to a decrease in bone resorption. Dmab is cleared by the reticuloendothelial system, with a half-life of approximately 26 days [131].

Dmab, as BPs, primarily increases theendocortical bone density, affecting the mineralization of endosteal resorption pits and thereby increasing the cortical thickness and reducing cortical porosity. Dmab decreases the risk of new VFs by 68% and non-VFs by 20% over 36 months [158].

In contrast to BPs, which have a long skeletal half-life with a persistence in anti-fracture benefits for a period of time after discontinuation, the concept of a “drug holiday” does not apply to other osteoporosis medications, which quickly lose their benefits after stopping. As an example, Dmab, which is not retained in the skeleton, should be followed by another medication, usually a BP, after discontinuation. Dmab is administered every 6 months; non-compliance with the dosing schedule can lead to a rebound increase in bone remodeling and bone loss, and an increased risk of multiple VFs [159]. The discontinuation of Dmab leads to an enhanced osteoclastogenesis and osteoblastogenesis, resulting in a loss in cortical thickness and trabecular bone volume along with a rapid acceleration of bone turnover and increased amount of unmineralized bone. Bone loss during the first year after Dmab discontinuation is approximately 5–11% at all skeletal sites [160]. BP-naïve patients may experience more bone loss after the discontinuation of Dmab in comparison with BP-treated patients [161]. BP-treated patients who have transitioned to Dmab have a greater BMD increase than those who continue BP therapy [162].

In the Fracture Reduction Evaluation of Denosumab in Osteoporosis Every 6 Months (FREEDOM) extension trial, Dmab was shown to be effective for up to 10 years, with an increase in BMD and sustained suppression of BTMs [163]. The duration of Dmab therapy plays a role in bone loss after switching from Dmab to a BP. Bone loss has not been described at the LS in patients who received one infusion of ZOL after ≤6 injections of Dmab (3-year therapy), in contrast to patients on longer-term treatment [164]. Of note, BTMs increase after stopping Dmab, regardless of the duration of the Dmab treatment [164]. The mechanisms for bone loss after the discontinuation of Dmab are unclear. Recent in vivo studies showed that osteoclasts can de-differentiate into non-resorbing daughter cells, “osteomorphs”, prior to being recycled as osteoclasts [165]. Osteomorphs can accumulate under the effect of RANKL inhibition as a reservoir, contributing to the bone turnover rebound when Dmab is stopped. Dmab does not delay fracture healing, even when administered around the time of the fracture [166].

***Teriparatide (TPTD) and abaloparatide*** are anabolic medications that are recombinant fragments of human PTH (1–34). TPTD is recombinant PTH (1–34); abaloparatide is a synthetic analog of PTH-related protein PTHrP (1–34). Abaloparatide has a 41% homology to PTH (1-34) and 76% homology to PTHrP (1–34).

Although a sustained PTH elevation in patients with hyperparathyroidism leads to an increased bone resorption and bone loss [167], the intermittent administration of TPTD stimulates bone remodeling and increases bone formation in excess of bone resorption [168]. Although TPTD also upregulates osteoclasts, the anabolic effect dominates [169]. New bone formation with TPTD is characterized by an increased cancellous bone volume and connectivity, improved trabecular morphology, and a shift toward a more plate-like structure, with an increased cortical bone thickness [170]. The effect of abaloparatide on bone metabolism is similar to TPTD. However, abaloparatide has a less pronounced activation effect on osteoclasts [171]. TPTD and abaloparatide increase the periosteal and endosteal perimeters, resulting in a larger, more structurally sound bone [172]. In general, bone effects of TPTD and abaloparatide are similar. TPTD and abaloparatide activate the type 1 PTH receptor (PTH1R), with a similar affinity for the R${}^{G}$ (GTPγS-sensitive) state of PTH1R, but TPTD has a higher affinity for R${}^{0}$ (GTPγS-insensitive) than abaloparatide, resulting in a prolonged cAMP signaling; AMP signaling is two-fold less with abaloparatide than teriparatide [173]. Abaloparatide has a faster dissociation time with PTH1R than TPTD, and, consequently, less of a bone resorptive effect [174].

In general, anabolic therapy is preferred over antiresorptive medications for optimizing outcomes of orthopedic surgery. Surgery delay and anabolic therapy have been recommended in patients considering elective spine surgery who have a low bone mass (T-score < −2.0), especially with a history of prior fragility fracture, in order to improve the skeletal health preoperatively [175]. It has been suggested by some that these medications should be started a minimum of 4 to 6 weeks prior to spine surgery and continued for up to 2 years [176]. Based on the recent recommendations, the anabolic therapy duration suggested is at least 2 months pre-operatively or up to 6-months pre-operatively for elective spine reconstructive surgery, with a postoperative duration of at least 8-months [2].

TPTD can shorten the postoperative time for fracture healing, reduce rates of delayed healing, and increase fusion rates, and may reduce non-union after BP-associated AFF [177,178].

Both anabolic medications increase the BMD in the spine and hip, with a better effect of abaloparatide at the total hip compared with TPTD [179]. Abaloparatide has been shown to increase TBS faster than TPTD [180]. Moreover, abaloparatide is associated with less of a decrease in 1/3 radius BMD (primarily cortical bone) than TPTD [181].

Based on an analysis of four prospective observational studies, TPTD reduces rates of clinical VFs, non-VFs, clinical fractures, and hip fractures by 62%, 43%, 50%, and 56%, respectively, with >6 mo of therapy compared with 0 to 6 mo [182]. Abaloparatide has been shown to reduce rates of VFs, non-VFs, and major osteoporotic fractures by 86%, 43%, and 70%, respectively, compared with placebo [183].

TPTD was initially limited to 24-month lifetime use due to an increase in the risk of osteosarcoma in rats. However, this restriction has been recently removed based on reviews of long-term post-marketing data, showing no evidence of an increase in osteosarcoma risk in humans [184,185].

It has been proposed that patients who can benefit from a longer duration of TPTD are ones with a very high fracture risk, unable to come off glucocorticoid therapy, with an elevated P1NP after two years of TPTD, or with multiple VFs at baseline but no fractures when on treatment [184]. Other proposed indications are adynamic bone disease and severe chronic obstructive pulmonary disease (COPD) with VFs, since there is a loss of approximately 8% of the vital capacity for each VF in this category of patients [184].

Due to reversible bone changes after anabolic medication discontinuation, follow-up antiresorptive therapy is essential. The European Study of Forsteo (EUROFORS) evaluated the effects of TPTD, raloxifene, and placebo for 1 year after 1 year of TPTD in postmenopausal women with severe osteoporosis. The LS BMD increased by 3.6% in patients who continued TPTD for a total of 2 years, remained stable for patients switched to raloxifene, and significantly decreased by 2.7% in patients transitioned to placebo [125]. At the TH, the change in BMD was +1.9%, +1.5%, and +0.3% with the transition to TPTD, raloxifene, and placebo, respectively. Changes in the FN were +2.6%, +1.6%, and +1.1%, respectively [125]. Hormone replacement therapy (HRT) can also prevent bone loss in the spine and hip for at least a year after TPTD discontinuation [186]. However, in premenopausal women, the discontinuation of TPTD after 2.0 ± 0.6 years of therapy led to a significant spine BMD loss of 4.8% but remained stable at the FN (−1.5 ± 4.2%), TH (−1.1 ± 3.7%), and 1/3 radius (+0.2 ± 2.5%) [187]. Antiresorptive treatment has been recommended for all premenopausal women with idiopathic osteoporosis after TPTD treatment, especially for patients who are >40 years old, and for patients with dramatic TPTD-related bone gain [187].

In general, treatment with Dmab or BPs is recommended after the discontinuation of any anabolic medication.

***Romosozumab (Rmab)*** is a humanized monoclonal antibody against sclerostin with the dual effect of stimulating bone modeling and inhibiting resorption, in contrast to other anabolic agents that stimulate remodeling via an increased formation and resorption of bone. Sclerostin, the Rmab target, is a glycoprotein produced by osteocytes that inhibits bone formation due to the downregulation of the Wnt pathway. Rmab binds to sclerostin and inhibits it activity [188], resulting in an increase in osteoblastic differentiation, proliferation, and survival. In the presence of Rmab, the Wnt signaling pathway is activated, leading to bone formation and BMD gain. Rmab systemic absorption after subcutaneous injection occurs via the lymphatic vessels to the blood compartment, with the elimination of monoclonal antibodies via protein catabolism. Partial elimination may occur at the target cells by endocytosis and intracellular degradation, which is concentration-dependent due to the saturation effect [189,190]. The role of hepatic and renal excretion in elimination is minor [191].

Bone histomorphometry has shown that two months of Rmab therapy results in an increase in the dynamic parameters of bone formation and a decrease in bone resorption compared with placebo [192]. Twelve months of therapy results in an increase in bone mass, trabecular thickness, and trabecular connectivity, with no significant change in cortical porosity [192]. A 3D microarchitecture assessment by μCT analysis demonstrated an improved trabecular connectivity.

In postmenopausal women, the dual effect of Rmab leads to a significant increase in BMD and a reduction in the fracture risk compared with alendronate and placebo [193,194]. Rmab 12 mo therapy led to a 73% lower relative risk of new VFs, 36% lower risk of clinical fractures, and no significant effect on non-VFs [194,195]. There was a difference in the non-VF reduction in Latin America vs. the rest of the world, with no treatment effect observed in Latin America vs. a 42% relative risk reduction in the rest-of-world population over 12 mo [195]. A recent meta-analysis found that Rmab increases the BMD more than TPTD [196].

With the sequence of Rmab for 1 year followed by Dmab for 1 year, patients from the FRActure study in postmenopausal woMen with ostEoporosis (FRAME) study achieved BMD T-score gains similar to those observed in patients from FREEDOM and FREEDOM Extension studies after 7 years of Dmab [116]. At the TH, a year of Rmab treatment produced BMD gains similar to those seen with 3 years of continuous Dmab treatment. Knowledge of the effect of Rmab on bone healing is limited. No effect on hip fracture healing has been demonstrated based on one study [197]. As for now, there is no evidence that would delay the initiation of osteoporosis medications after a fracture or after bone-related surgery.

Changes in BMD related to the medication sequence are described in Table 6.

Anti-osteoporosis medications have possible side effects. However, serious side effects are rare; these include AFF, described in a separate section, and osteonecrosis of the jaw (ONJ). ONJ risk factors include prolonged BP use, periodontitis, dental procedures, poor oral hygiene, the use of removable apparatus, glucocorticoid use, and an age of 65 years and older [205,206,207,208]. Tooth extraction is one of the most common immediate triggers of ONJ. However, approximately 14% of ONJ occur spontaneously [209,210]. ONJ can be seen with BP and Dmab use, and may occur in patients with no osteoporosis therapy. The rate is higher in patients treated for cancer, who are typically treated with higher doses and an increased frequency of BP or Dmab compared to patients treated for osteoporosis. The prevalence of ONJ in oral BP users has been described as ranging from 0.001% to 0.01% (1/10,000 to 1/100,000 patient-years) [19]. The rate is 0.017% after IV BP use for 3 years [211]. The risk of ONJ may be higher with Dmab vs. oral or IV BPs. Dmab-induced ONJ was reported as 5.2 per 10,000 patient/years (0.0052%) [163]. Among 3068 patients treated with BPs or Dmab for OP, ONJ developed in 12 patients on Dmab and 5 patients in BPs. The ONJ incidence per 10,000 observed patient-years was 28.3 for Dmab vs. 4.5 for BP-treated patients. However, 9 of 12 patients treated with Dmab had previous history of BP use [212].

A few cases of ONJ have been described after Rmab use: in FRAME, two patients developed ONJ (0.06%) after completion of the 12 mo treatment with Rmab. Both patients had dental issues, with 1 having ill-fitting dentures and the other having a tooth extraction resulting in osteomyelitis after receiving follow-up treatment with Dmab [194]. TPTD and abaloparatide are not associated with ONJ. Moreover, TPTD may improve the rate of medication-related ONJ resolution [213].

## 8. Specifics of Fracture Healing and Treatment with AFF

AFF is a very rare complication of BP therapy, with the benefits of treatment far exceeding the risks in appropriately selected patients [214]. Patients 65–84 years of age have a higher rate of AFF compared with younger or older patients, while the incidence of “typical” hip fractures increases with aging. Asians are predisposed to AFF in comparison with white women. Other risk factors for AFF include a higher body weight, shorter height, and one or more years of glucocorticoid use. BMD has not been associated with the AFF risk [214].

Reports suggest a strong relationship between AFF and BP treatment [215,216,217,218,219]. There is a suggestion that this may be due to the prolonged suppression of bone remodeling with a reduced osteoclast activity [219,220]. There is an assumption that less effective remodeling may cause the mineral density of bones to increase and the bone matrix to become more homogeneous [219,221]. This may, in turn, cause an increase in the mineral-to-matrix ratio; as a result, bones become brittle and more susceptible to crack formation and propagation [219].

Published research results suggest that some features of femoral geometry and changes in bone microstructure caused by prolonged BP treatment may contribute to AFF [222]. There are various factors causing AFF at macro- and microscale mechanisms. Tensile stresses in the lateral femoral cortex may increase due to certain features of femoral geometry contributing to a higher AFF risk [222]. Greater tensile stresses in bones may be caused by femur curvature. Thus, individuals with bowed femurs are at a higher risk of bending stresses, which may lead to fractures of the lateral femoral cortex [223].

AFF begins as a stress fracture on the lateral cortex of the proximal cortex, where there is a high tensile load due to the bending [223]. The region adjacent to the fracture line contains many resorption cavities and channels. Most of them are oriented perpendicular to the fracture plane, but some channels run transversely [219,224,225]. Osteoclasts are frequently present in resorption cavities located in close proximity to the fracture line and are less frequently found further away. Approximately 25% of the osteoclasts adjacent to superficial resorption cavities are giant cells containing pyknotic nuclei [225,226]. A morphometric assessment of the process of reversing resorption to formation demonstrated that BPs hamper the onset of bone formation after resorption [225,227].

AFF definition criteria were developed by the American Society for Bone and Mineral Research in 2010 and revised in 2013 [218]. To categorize a fracture as AFF, at least four of the five major features listed below must be present. Minor features listed below are not required but, when present, can corroborate the AFF diagnosis [218].

***Major features***

1. The fracture is associated with minimal or no trauma, such as in a fall from standing height or lower;
2. The fracture line originates at the lateral cortex and is substantially transverse in its orientation, although it may become oblique as it progresses medially across the femur;
3. Complete fractures extend through both cortices and may be associated with a medial spike; incomplete fractures involve only the lateral cortex;
4. The fracture is noncomminuted or minimally comminuted;
5. Localized periosteal or endosteal thickening of the lateral cortex is present at the fracture site (‘‘beaking’’ or ‘‘flaring’’).

***Minor features***

1. A generalized increase in cortical thickness of the femoral diaphyses;
2. Unilateral or bilateral prodromal symptoms, such as dull or aching pain in the groin or thigh;
3. Bilateral incomplete or complete femoral diaphysis fractures;
4. Delayed fracture healing.

An analysis of women with a history of BP use showed an increase in the incidence of AFF with a longer duration of therapy, from 0.1 (<3 mo on BP), 0.6 (<3 years on BP), 2.5 (<5 years on BP), and 6.0 (<8 years on BP) to 13.1 per 10,000 person-years (>8 years on BP) [214]. Of note, the risk of patients who have never been on BP-therapy was reported as 0.10 per 10,000 person-years [214]. Bilateral AFFs were reported in 28-44% in AFF patients [228,229]. There is a rapid decrease in AFF by 48% 3–15 months after the discontinuation of BP and a 74–79% risk reduction in subsequent years.

The absolute risk of AFF is very low in Dmab and raloxifene users [230]. Only three AFF cases were described with Rmab, with two occurring during the alendronate treatment phase. Since Rmab has only recently been approved (in 2019), more time is needed to evaluate the risk for AFF. Described AFF cases in patients on TPTD had previous BP exposure. No cases of AFF were reported in patients on abaloparatide.

Based on the recently proposed AFF management recommendations [230], in patients with unilateral or non-surgically treated AFF who are at a high risk for fragility fractures, treatment with TPTD or abaloparatide should be considered. However, transitioning from Dmab to TPTD may be followed by sustained BMD loss at the hip [200]. When TPTD or abaloparatide cannot be used, transitioning to Rmab, raloxifene, estrogen, or calcitonin should be considered. For AFF patients with a low risk of fragility fractures who received more than two injections of Dmab, a short course of BP or raloxifene can be given. If AFF is surgically treated, a short course of TPTD (3–6 months) can be considered; however, the data for the benefits are weak. Monitoring with imaging 1–2 years after AFF is recommended to ensure healing and assess for contralateral AFF [230].

In conclusion, there are no FDA-approved medications for enhancing fracture healing or the outcomes of skeletal surgery. However, there is accumulating evidence supporting the use of pharmacologic therapy in patients with poor bone health before and/or after bone surgery. There is no evidence that anti-osteoporosis medications delay healing after bone surgery.

## Figures and Tables

**Table 4:** Effect of anti-osteoporosis medications treatment for 12–18 months on BMD.

Therapy	ROI	Change vs. Baseline, %	Treatment, Months	Patient Population	Ref.
Teriparatide	LS	10.04 ± 5.23%	12	Japanese W and men	[34]
Teriparatide	LS	6.9%	12	Postmenopausal W, 55–85 years	[115]
Teriparatide	FN	2.01 ± 4.63%	12	Japanese W and men	[34]
Teriparatide	TH	2.72 ± 4.04%	12	Japanese W and men	[34]
Teriparatide	TH	0.8%	12	Postmenopausal W, 55–85 years	[115]
Romosozumab (FRAME)	LS	BMD increase: 96% of patients ≥ 3% 89% > 6%, 68% ≥ 10%,	12	Postmenopausal W	[116]
Romosozumab (ARCH)	LS	BMD increase: 14.7%	12	Postmenopausal W	[117]
Romosozumab	LS	9.1%	12	Postmenopausal W, 55–85 years	[118]
Romosozumab	LS	12.3%	12	Postmenopausal W, 55–85 years	[115]
Romosozumab (FRAME)	TH/FN	BMD increase: 78% of patients ≥ 3%, 47% > 6%, 16% ≥10%	12	Postmenopausal W	[116]
Romosozumab	FN	3.9%	12	Postmenopausal W, 55–85 yo	[118]
Romosozumab	TH	4.6%	12	Postmenopausal W, 55–85 yo	[118]
Romosozumab	TH	3.9%	12	Postmenopausal W, 55–85 yo	[115]
Zoledronic acid	LS	3.93 ± 0.34%	12	Chinese Postmenopausal W	[119]
Zoledronic acid	FN	2.69 ± 0.46%	12	Chinese Postmenopausal W	[119]
Zoledronic acid	TH	2.81 ± 0.32%	12	Chinese Postmenopausal W	[119]
Alendronate (ARCH)	LS	4.4%	12	Postmenopausal W	[117]
Alendronate or Zoledronic acid	LS	4.5% ± 11.6	At least 12	Postmenopausal W, 53–66 years	[120]
Alendronate or Zoledronic acid	FN	3.8% ± 7.3	At least 12	Postmenopausal W, 53–66 years	[120]
Denosumab	LS	5.4%	12	Postmenopausal W, >55 yeras	[121]
Denosumab	LS	9.03% ± 11.3	At least 12	Postmenopausal W, 53–66 years	[120]
Denosumab	TH	3.1%	12	Postmenopausal W, >55 years	[121]
Denosumab	FN	2.7%	12	Postmenopausal W, >55 years	[121]
Denosumab	FN	8.7% ± 8.5	At least 12	Postmenopausal W, 53–66 years	[120]
Abaloparatide (ACTIVE)	LS	11.2%	18	Postmenopausal W, 49–86 years	[122]
Abaloparatide (ACTIVE)	LS	12.1%	18	Postmenopausal W, >80 years	[123]
Abaloparatide (ACTIVE)	LS	7.81%	18	Postmenopausal W, <65 years	[124]
Abaloparatide (ACTIVE)	FN	3.6%	18	Postmenopausal W, 49–86 years	[122]
Abaloparatide (ACTIVE)	FN	3.6%	18	Postmenopausal W, >80 years	[123]
Abaloparatide (ACTIVE)	FN	2.71%	18	Postmenopausal W, <65 years	[124]
Abaloparatide (ACTIVE)	TH	4.18%	18	Postmenopausal W, 49–86 years	[122]
Abaloparatide (ACTIVE)	TH	3.9%	18	Postmenopausal W, >80 years	[123]
Abaloparatide (ACTIVE)	TH	3.2%	18	Postmenopausal W, <65 years	[124]

BMD—bone mineral density; FN—femoral neck LS—lumbar spine; ROI—region of interest; TH—total hip; W—women.

**Table 5:** Effect of anti-osteoporosis medications treatment for 24–36 months on BMD.

Therapy	ROI	Change vs. Baseline, %	Treatment, Months	Patient Population	Ref.
Teriparatide	LS	13.42 ± 6.12%	24	Japanese W and men	[34]
Teriparatide	LS	10.70%	24	Postmenopausal W	[125]
Teriparatide	LS	14.2 ± 8.1	24; 2 patients −18	Premenopausal W	[126]
Teriparatide	FN	3.26 ± 4.25%	24	Japanese W and men	[34]
Teriparatide	FN	3.50%	24	Postmenopausal W	[125]
Teriparatide	FN	5.1 ± 5.2%	24; 2 patients −18	Premenopausal W	[126]
Teriparatide	TH	3.67 ± 3.98%	24	Japanese W and men	[34]
Teriparatide	TH	2.50%	24	Postmenopausal W	[125]
Teriparatide	TH	5.3 ± 4.3%	24; 2 patients −18	Premenopausal W	[126]
Zoledronic acid	LS	5.71 ± 0.35%	24	Chinese Postmenopausal W	[119]
Zoledronic acid	FN	3.36 ± 0.60%	24	Chinese Postmenopausal W	[119]
Zoledronic acid	TH	3.7 ± 0.46%	24	Chinese Postmenopausal W	[119]
Alendronate (ARCH)	LS	7.40%	24	Postmenopausal W	[117]
Denosumab	LS	9.20%	36	Postmenopausal W, 60–90 years	[127]
Denosumab	TH	6.00%	36	Postmenopausal W, 60–90 years	[127]

BMD—bone mineral density; FN—femoral neck LS—lumbar spine; ROI—region of interest; TH—total hip; W—women.

**Table 6:** Change in BMD based on sequence of antiosteoporosis medication use.

Initial Drug	Second Drug	Effect on BMD	Reference
BPs	TPTD	BP-pretreated vs. BP-naïve patients started on teriparatide: The greatest mean increase in BMD: LS: BP-naïve: 15.46% (11.60–19.31%) at 18 mo BP-pretreated 11.20% (8.56–13.85%) at 24 mo FN: BP-naive, 5.16% [2.32–8.00%] at 24 mo BP-pretreated: 2.22% [0.72–3.72%] at 24 mo TH: BP-naive group: BMD decreased at 6 mo (NS), then increased significantly at 12, 18, and 24 mo; BP-pretreated group: BMD decreased slightly from baseline at 6, 12, and 18 mo, then increased from baseline at 24 mo (NS). The greatest increase observed in the BP-naive group: 4.46% [0.98–7.94%] at 24 mo	[198]
ALN for at least 6 mo	Dmab 12 mo	Switch to Dmab for 12 mo: vs. continued ALN: LS: 3.03% vs. 1.85% (*p* < 0.05) TH: 1.9% vs. 1.05% (NS) FN and 1/3 radius: significantly higher BMD for Dmab	[162]
Oral BP at least 3 yr and ALN 1 yr	Rmab 12 mo TPTD 12 mo	Effect of Rmab vs. TPTD for 12 mo after BPs: LS: 9.8% vs. 5.4% (*p* < 0.05) TH: 2.6% vs. −0.6% (*p* < 0.05) FN: 3.2% vs. −0.2% (*p* < 0.05)	[199]
Dmab 24 mo DATA-Switch study	TPTD 24 mo	Postmenopausal women 24 months of teriparatide + 24 months of Dmab: LS: decreased over first 6 mo followed by mean net 48-month increase of 14.0 ± 6.7% Increase in Dmab only: 4.8 ± 5.6% TH: progressively decreased between 24–36 mo Change after transitioning: −0.7 ± 3.1, FN: transient bone loss occurring between 24–36 mo, net 48-month increase of 4.9 ± 6.0% Change after transitioning: 1.2 ± 4.9% 1/3 forearm: net 48 mo decrease of −1.8 ± 5.9%	[200]
Dmab 12 mo	Rmab 12 mo	LS +11.5% TH +3.8% FN +3.2%	[118]
Dmab 12 mo	Rmab 12 mo (Second course)	LS +2.3% (95% CI 0.3, 4.4) TH −0.1% (95% CI −1.2, 0.9) FN +0.8% (95% CI −0.3, 2.0)	[201]
Dmab 12 mo DAPS study	BPs (ALN) 12 mo	24 mo BMD change (Dmab 12 mo + ALN 12 mo): LS +5.9% TH +3.6% FN +2.5% BMD gain in ALN only LS +0.5% TH + 0.5% FN −0.2%	[121]
Dmab 12 mo	ZOL 1 dose 12 and 24 mo	LS 1.7% ± 1.1% at 12 mo LS 0.1% ± 1.2% at 24 mo	[202]
Rmab 12 mo ARCH study	BPs (ALN) 12 mo	LS: net 24-month increase of 17%	[117]
Rmab 12 mo FRAME Extension study	Dmab 12 mo and 24 mo	Differences in BMD increases from baseline Rmab-to-Dmab vs. placebo-to-Dmab 12 and 24 mo LS: 11.8% and 10.5% TH: 5.3% and 5.2% FN: 4.9% and 4.8% BMD after Rmab → 12 and 24 mo on Dmab LS: 13.1% → 16.6% → 18.1% TH:6% → 8.5% → 9.4% FN: 5.5% → 7.3% → 8.2%	[203]
TPTD 24 mo DATA-Switch study	Dmab 24 mo	Postmenopausal women 24 months of teriparatide + 24-month of Dmab: LS: net 48-month increase of 18.3 ± 8.5%, Change after transitioning: +8.6 ± 5.0% TH: net 48-month increase of 6.6 ± 3.3% Change after transitioning: +4.7 ± 2.6% FN: net 48-month increase of 8.3 ± 5.6% Change after transitioning: 5.6 ± 4.5% 1/3 forearm: net 48 mo decrease of 0.0 ± 2.9%	[200]
TPTD 24 mo	Dmab 12 and 24 mo	Premenopausal women with IOP 24 months of teriparatide + 24 months of Dmab: BMD increased by: LS: 21.9 ± 7.8% TH: 9.8 ± 4.6% FN: 9.5 ± 4.7% BMD increase after 12 months and 24 mo of Dmab after Teriparatide for 24 mo: LS: 5.2 ± 2.6% and 6.9 ± 2.6%, TH: 2.9 ± 2.4% and 4.6 ± 2.8% FN: 3.0 ± 3.8% and 4.7 ± 4.9%	[126]
TPTD 12 mo EUROFORS	Raloxifene 12 mo	BMD change after 24 mo of Raloxifene after Teriparatide for 12 mo: LS: no change TH: 2.3% FN: 3.1%	[125]
TPTD 24 mo	ALN or Dmab	ALN, 12 mo: LS: +1.3 ± 5.1% FN: +0.7 ± 4.6% Dmab, 12 mo: LS: +4.3 ± 3.5% FN: +1.4 ± 3.4%	[204]

ALN—alendronate; BMD—bone mineral density; BPs—bisphosphonates; Dmab—denosumab; FN—femoral neck; LS—lumbar spine; NS—not significant, Rmab—romosozumab, TPTD—teriparatide; ROI—region of interest; TH—total hip.

## Data Availability

Not applicable.

## References

[1] Hak D.J. (2018). The biology of fracture healing in osteoporosis and in the presence of anti-osteoporotic drugs. Injury.

[2] Sardar Z.M., Coury J.R., Cerpa M., DeWald C.J., Ames C.P., Shuhart C., Watkins C., Polly D.W., Dirschl D.R., Klineberg E.O. (2022). Best Practice Guidelines for Assessment and Management of Osteoporosis in Adult Patients Undergoing Elective Spinal Reconstruction. Spine.

[3] Lafuente-Gracia L., Borgiani E., Nasello G., Geris L. (2021). Towards in silico Models of the Inflammatory Response in Bone Fracture Healing. Front. Bioeng Biotechnol..

[4] Giannoudis P.V., Einhorn T.A., Marsh D. (2007). Fracture healing: The diamond concept. Injury.

[5] Sheen J.R., Garla V.V. (2021). Fracture Healing Overview. StatPearls.

[6] Marsell R., Einhorn T.A. (2011). The biology of fracture healing. Injury.

[7] Schindeler A., McDonald M.M., Bokko P., Little D.G. (2008). Bone remodeling during fracture repair: The cellular picture. Semin. Cell Dev. Biol..

[8] Barnes G.L., Kostenuik P.J., Gerstenfeld L.C., Einhorn T.A. (1999). Growth factor regulation of fracture repair. J. Bone Miner. Res..

[9] Carano R.A., Filvaroff E.H. (2003). Angiogenesis and bone repair. Drug Discov. Today.

[10] Tsiridis E., Upadhyay N., Giannoudis P. (2007). Molecular aspects of fracture healing: Which are the important molecules?. Injury.

[11] Cox G., Einhorn T.A., Tzioupis C., Giannoudis P.V. (2010). Bone-turnover markers in fracture healing. J. Bone Jt. Surg. Br..

[12] Kangari P., Talaei-Khozani T., Razeghian-Jahromi I., Razmkhah M. (2020). Mesenchymal stem cells: Amazing remedies for bone and cartilage defects. Stem Cell Res. Ther..

[13] Bone Health & Osteoporosis Foundation What is Osteoporosis and What Causes It?. https://www.nof.org/patients/what-is-osteoporosis/.

[14] Pesce V., Speciale D., Sammarco G., Patella S., Spinarelli A., Patella V. (2009). Surgical approach to bone healing in osteoporosis. Clin. Cases Miner Bone Metab..

[15] Doll B., Tegtmeier F., Koch H., Acarturk O., Holliger J. (2003). Declino cellulare e molecolare nella guarigione ossea con l’avanzare dell’età. Tec. Chir. Ortop..

[16] Verschueren S., Gielen E., O’Neill T.W., Pye S.R., Adams J.E., Ward K.A., Wu F.C., Szulc P., Laurent M., Claessens F. (2013). Sarcopenia and its relationship with bone mineral density in middle-aged and elderly European men. Osteoporos. Int..

[17] Maurel D.B., Jahn K., Lara-Castillo N. (2017). Muscle-Bone Crosstalk: Emerging Opportunities for Novel Therapeutic Approaches to Treat Musculoskeletal Pathologies. Biomedicines.

[18] Hamrick M.W. (2012). The skeletal muscle secretome: An emerging player in muscle-bone crosstalk. Bonekey Rep..

[19] Farr J.N., Fraser D.G., Wang H., Jaehn K., Ogrodnik M.B., Weivoda M.M., Drake M.T., Tchkonia T., LeBrasseur N.K., Kirkland J.L. (2016). Identification of Senescent Cells in the Bone Microenvironment. J. Bone Miner. Res..

[20] Hao Y., Ma Y., Wang X., Jin F., Ge S. (2012). Short-term muscle atrophy caused by botulinum toxin-A local injection impairs fracture healing in the rat femur. J. Orthop. Res..

[21] Kushchayeva Y., Lewiecki E.M. (2021). Osteoporosis management with focus on spine. Image Guided Interventions of the Spine: Principles and Clinical Applications.

[22] Sousa C.P., Dias I.R., Lopez-Peña M., Camassa J.A., Lourenço P.J., Judas F.M., Gomes M.E., Reis R.L. (2015). Bone turnover markers for early detection of fracture healing disturbances: A review of the scientific literature. An. Acad. Bras Cienc..

[23] Pan C., Liu X., Li T., Wang G., Sun J. (2018). Kinetic of bone turnover markers after osteoporotic vertebral compression fractures in postmenopausal female. J. Orthop. Surg. Res..

[24] Szulc P., Naylor K., Hoyle N.R., Eastell R., Leary E.T., for the National Bone Health Alliance Bone Turnover Marker Project (2017). Use of CTX-I and PINP as bone turnover markers: National Bone Health Alliance recommendations to standardize sample handling and patient preparation to reduce pre-analytical variability. Osteoporos. Int..

[25] Cosman F., de Beur S.J., LeBoff M.S., Lewiecki E.M., Tanner B., Randall S., Lindsay R. (2014). Clinician’s Guide to Prevention and Treatment of Osteoporosis. Osteoporos. Int..

[26] Ivaska K.K., Gerdhem P., Akesson K., Garnero P., Obrant K.J. (2007). Effect of fracture on bone turnover markers: A longitudinal study comparing marker levels before and after injury in 113 elderly women. J. Bone Miner. Res..

[27] Hannon R., Eastell R. (2000). Preanalytical variability of biochemical markers of bone turnover. Osteoporos. Int..

[28] Veitch S., Findlay S., Hamer A., Blumsohn A., Eastell R., Ingle B. (2006). Changes in bone mass and bone turnover following tibial shaft fracture. Osteoporos. Int..

[29] Lorentzon M., Branco J., Brandi M.L., Bruyère O., Chapurlat R., Cooper C., Cortet B., Diez-Perez A., Ferrari S., Gasparik A. (2019). Algorithm for the Use of Biochemical Markers of Bone Turnover in the Diagnosis, Assessment and Follow-Up of Treatment for Osteoporosis. Adv. Ther..

[30] Vasikaran S., Eastell R., Bruyere O., Foldes A., Garnero P., Griesmacher A., McClung M., Morris H.A., Silverman S., Trenti T. (2011). Markers of bone turnover for the prediction of fracture risk and monitoring of osteoporosis treatment: A need for international reference standards. Osteoporos. Int..

[31] Ohishi T., Takahashi M., Kushida K., Hoshino H., Tsuchikawa T., Naitoh K., Inoue T. (1998). Changes of biochemical markers during fracture healing. Arch. Orthop. Trauma Surg..

[32] Hlaing T.T., Compston J.E. (2014). Biochemical markers of bone turnover - uses and limitations. Ann. Clin. Biochem..

[33] Park S.Y., Ahn S.H., Yoo J.I., Chung Y.J., Jeon Y.K., Yoon B.H., Kim H.Y., Lee S.H., Lee J., Hong S. (2019). Position Statement on the Use of Bone Turnover Markers for Osteoporosis Treatment. JBM.

[34] Miyauchi A., Matsumoto T., Sugimoto T., Tsujimoto M., Warner M.R., Nakamura T. (2010). Effects of teriparatide on bone mineral density and bone turnover markers in Japanese subjects with osteoporosis at high risk of fracture in a 24-month clinical study: 12-month, randomized, placebo-controlled, double-blind and 12-month open-label phases. Bone.

[35] Lenora J., Norrgren K., Thorsson O., Wollmer P., Obrant K.J., Ivaska K.K. (2009). Bone turnover markers are correlated with total skeletal uptake of 99mTc-methylene diphosphonate (99mTc-MDP). BMC Med. Phys..

[36] Chew C.K., Clarke B.L. (2017). Biochemical Testing Relevant to Bone. Endocrinol. Metab. Clin. N. Am..

[37] Bover J., Ureña-Torres P., Cozzolino M., Rodríguez-García M., Gómez-Alonso C. (2021). The Non-invasive Diagnosis of Bone Disorders in CKD. Calcif. Tissue Int..

[38] Jain S., Camacho P. (2018). Use of bone turnover markers in the management of osteoporosis. Curr. Opin. Endocrinol. Diabetes Obes..

[39] Greenblatt M.B., Tsai J.N., Wein M.N. (2017). Bone Turnover Markers in the Diagnosis and Monitoring of Metabolic Bone Disease. Clin. Chem..

[40] Galbusera F., Volkheimer D., Reitmaier S., Berger-Roscher N., Kienle A., Wilke H.J. (2015). Pedicle screw loosening: A clinically relevant complication?. Eur. Spine J..

[41] Park S.B., Chung C.K. (2011). Strategies of spinal fusion on osteoporotic spine. J. Korean Neurosurg. Soc..

[42] Chin D.K., Park J.Y., Yoon Y.S., Kuh S.U., Jin B.H., Kim K.S., Cho Y.E. (2007). Prevalence of osteoporosis in patients requiring spine surgery: Incidence and significance of osteoporosis in spine disease. Osteoporos. Int..

[43] Lubelski D., Choma T.J., Steinmetz M.P., Harrop J.S., Mroz T.E. (2015). Perioperative Medical Management of Spine Surgery Patients With Osteoporosis. Neurosurgery.

[44] Morita A., Kobayashi N., Choe H., Tezuka T., Higashihira S., Inaba Y. (2021). Preoperative factors predicting the severity of BMD loss around the implant after Total hip Arthroplasty. BMC Musculoskelet. Disord..

[45] Aro H.T., Alm J.J., Moritz N., Mäkinen T.J., Lankinen P. (2012). Low BMD affects initial stability and delays stem osseointegration in cementless total hip arthroplasty in women: A 2-year RSA study of 39 patients. Acta Orthop..

[46] Virtama P. (1960). Uneven distribution of bone minerals and covering effect of non-mineralized tissue as reasons for impaired detectability of bone density from roentgenograms. Ann. Med. Intern. Fenn..

[47] McCullagh C.D., McCoy K., Crawford V.L., Taggart H. (2003). How reliable is a radiological report in osteoporosis in diagnosing low bone density?. Ulster Med. J..

[48] Naylor K.L., McArthur E., Leslie W.D., Fraser L.A., Jamal S.A., Cadarette S.M., Pouget J.G., Lok C.E., Hodsman A.B., Adachi J.D. (2014). The three-year incidence of fracture in chronic kidney disease. Kidney Int..

[49] Kwon Y.E., Choi H.Y., Kim S., Ryu D.R., Oh H.J. (2019). Fracture risk in chronic kidney disease: A Korean population-based cohort study. Kidney Res. Clin. Pract..

[50] Kim C.W., Kim H.J., Lee C.R., Wang L., Rhee S.J. (2020). Effect of chronic kidney disease on outcomes of total joint arthroplasty: A meta-analysis. Knee Surg. Relat. Res..

[51] Chou T.A., Ma H.H., Tsai S.W., Chen C.F., Wu P.K., Chen W.M. (2021). Dialysis patients have comparable results to patients who have received kidney transplant after total joint arthroplasty: A systematic review and meta-analysis. EFORT Open Rev..

[52] Popat R., Ali A.M., Holloway I.P., Sarraf K.M., Hanna S.A. (2021). Outcomes of total hip arthroplasty in haemodialysis and renal transplant patients: Systematic review. Hip Int..

[53] Iimori S., Mori Y., Akita W., Kuyama T., Takada S., Asai T., Kuwahara M., Sasaki S., Tsukamoto Y. (2012). Diagnostic usefulness of bone mineral density and biochemical markers of bone turnover in predicting fracture in CKD stage 5D patients—A single-center cohort study. Nephrol. Dial. Transplant..

[54] Kidney Disease: Improving Global Outcomes (KDIGO) CKD-MBD Update Work Group (2017). KDIGO 2017 Clinical Practice Guideline Update for the Diagnosis, Evaluation, Prevention, and Treatment of Chronic Kidney Disease-Mineral and Bone Disorder (CKD-MBD). Kidney Int. Suppl..

[55] West S.L., Lok C.E., Langsetmo L., Cheung A.M., Szabo E., Pearce D., Fusaro M., Wald R., Weinstein J., Jamal S.A. (2015). Bone mineral density predicts fractures in chronic kidney disease. J. Bone Miner. Res..

[56] Messina C., Bandirali M., Sconfienza L.M., D’Alonzo N.K., Di Leo G., Papini G.D., Ulivieri F.M., Sardanelli F. (2015). Prevalence and type of errors in dual-energy X-ray absorptiometry. Eur. Radiol..

[57] Albano D., Agnollitto P.M., Petrini M., Biacca A., Ulivieri F.M., Sconfienza L.M., Messina C. (2021). Operator-Related Errors and Pitfalls in Dual Energy X-Ray Absorptiometry: How to Recognize and Avoid Them. Acad. Radiol..

[58] Martineau P., Bazarjani S., Zuckier L.S. (2015). Artifacts and Incidental Findings Encountered on Dual-Energy X-Ray Absorptiometry: Atlas and Analysis. Semin. Nucl. Med..

[59] Morgan S.L., Prater G.L. (2017). Quality in dual-energy X-ray absorptiometry scans. Bone.

[60] Gupta A., Upadhyaya S., Patel A., Fogel H.A., Cha T., Schwab J., Bono C., Hershman S. (2020). DEXA sensitivity analysis in patients with adult spinal deformity. Spine J..

[61] Silva B.C., Leslie W.D., Resch H., Lamy O., Lesnyak O., Binkley N., McCloskey E.V., Kanis J.A., Bilezikian J.P. (2014). Trabecular bone score: A noninvasive analytical method based upon the DXA image. J. Bone Miner. Res..

[62] Krohn K., Schwartz E.N., Chung Y.S., Lewiecki E.M. (2019). Dual-energy X-ray Absorptiometry Monitoring with Trabecular Bone Score: 2019 ISCD Official Position. J. Clin. Densitom..

[63] Pothuaud L., Barthe N., Krieg M.A., Mehsen N., Carceller P., Hans D. (2009). Evaluation of the potential use of trabecular bone score to complement bone mineral density in the diagnosis of osteoporosis: A preliminary spine BMD-matched, case-control study. J. Clin. Densitom..

[64] Winzenrieth R., Dufour R., Pothuaud L., Hans D. (2010). A retrospective case-control study assessing the role of trabecular bone score in postmenopausal Caucasian women with osteopenia: Analyzing the odds of vertebral fracture. Calcif. Tissue Int..

[65] Rabier B., Heraud A., Grand-Lenoir C., Winzenrieth R., Hans D. (2010). A multicentre, retrospective case-control study assessing the role of trabecular bone score (TBS) in menopausal Caucasian women with low areal bone mineral density (BMDa): Analysing the odds of vertebral fracture. Bone.

[66] Lamy O., Metzger M., Krieg M.A., Aubry-Rozier B., Stoll D., Hans D. (2011). [OsteoLaus: Prediction of osteoporotic fractures by clinical risk factors and DXA, IVA and TBS]. Rev. Med. Suisse.

[67] Hans D., Goertzen A.L., Krieg M.A., Leslie W.D. (2011). Bone microarchitecture assessed by TBS predicts osteoporotic fractures independent of bone density: The Manitoba study. J. Bone Miner. Res..

[68] Diez-Perez A., Brandi M.L., Al-Daghri N., Branco J.C., Bruyère O., Cavalli L., Cooper C., Cortet B., Dawson-Hughes B., Dimai H.P. (2019). Radiofrequency echographic multi-spectrometry for the in-vivo assessment of bone strength: State of the art-outcomes of an expert consensus meeting organized by the European Society for Clinical and Economic Aspects of Osteoporosis, Osteoarthritis and Musculoskeletal Diseases (ESCEO). Aging Clin. Exp. Res..

[69] Casciaro S., Peccarisi M., Pisani P., Franchini R., Greco A., De Marco T., Grimaldi A., Quarta L., Quarta E., Muratore M. (2016). An Advanced Quantitative Echosound Methodology for Femoral Neck Densitometry. Ultrasound Med. Biol..

[70] Conversano F., Franchini R., Greco A., Soloperto G., Chiriacò F., Casciaro E., Aventaggiato M., Renna M.D., Pisani P., Di Paola M. (2015). A novel ultrasound methodology for estimating spine mineral density. Ultrasound Med. Biol..

[71] Di Paola M., Gatti D., Viapiana O., Cianferotti L., Cavalli L., Caffarelli C., Conversano F., Quarta E., Pisani P., Girasole G. (2019). Radiofrequency echographic multispectrometry compared with dual X-ray absorptiometry for osteoporosis diagnosis on lumbar spine and femoral neck. Osteoporos. Int..

[72] Adami G., Arioli G., Bianchi G., Brandi M.L., Caffarelli C., Cianferotti L., Gatti D., Girasole G., Gonnelli S., Manfredini M. (2020). Radiofrequency echographic multi spectrometry for the prediction of incident fragility fractures: A 5-year follow-up study. Bone.

[73] Bojinca V.C., Popescu C.C., Decianu R.D., Dobrescu A., Balanescu S.M., Balanescu A.R., Bojinca M. (2019). A novel quantitative method for estimating bone mineral density using B-mode ultrasound and radiofrequency signals-a pilot study on patients with rheumatoid arthritis. Exp. Ther. Med..

[74] Cortet B., Dennison E., Diez-Perez A., Locquet M., Muratore M., Nogues X., Ovejero Crespo D., Quarta E., Brandi M.L. (2021). Radiofrequency Echographic Multi Spectrometry (REMS) for the diagnosis of osteoporosis in a European multicenter clinical context. Bone.

[75] Valentina Anna D., Maria Luisa B., Greta C., Sergio C., Delia C., Francesco C., Pasquo Elvira D., Stefano G., Fiorella Anna L., Paola P. (2021). First assessment of bone mineral density in healthy pregnant women by means of Radiofrequency Echographic Multi Spectrometry (REMS) technology. Eur. J Obstet. Gynecol. Reprod. Biol..

[76] Jang S., Graffy P.M., Ziemlewicz T.J., Lee S.J., Summers R.M., Pickhardt P.J. (2019). Opportunistic Osteoporosis Screening at Routine Abdominal and Thoracic CT: Normative L1 Trabecular Attenuation Values in More than 20 000 Adults. Radiology.

[77] (2019). 2019 ISCD Official Positions Adult. https://iscd.org/learn/official-positions/adult-positions/.

[78] Lee S.Y., Kwon S.S., Kim H.S., Yoo J.H., Kim J., Kim J.Y., Min B.C., Moon S.J., Sung K.H. (2015). Reliability and validity of lower extremity computed tomography as a screening tool for osteoporosis. Osteoporos. Int..

[79] Pickhardt P.J., Pooler B.D., Lauder T., del Rio A.M., Bruce R.J., Binkley N. (2013). Opportunistic screening for osteoporosis using abdominal computed tomography scans obtained for other indications. Ann. Intern. Med..

[80] Buckens C.F., Dijkhuis G., de Keizer B., Verhaar H.J., de Jong P.A. (2015). Opportunistic screening for osteoporosis on routine computed tomography? An external validation study. Eur. Radiol..

[81] Sadineni R.T., Pasumarthy A., Bellapa N.C., Velicheti S. (2015). Imaging Patterns in MRI in Recent Bone Injuries Following Negative or Inconclusive Plain Radiographs. J. Clin. Diagn. Res..

[82] Shen W., Chen J., Punyanitya M., Shapses S., Heshka S., Heymsfield S.B. (2007). MRI-measured bone marrow adipose tissue is inversely related to DXA-measured bone mineral in Caucasian women. Osteoporos. Int..

[83] Shen W., Scherzer R., Gantz M., Chen J., Punyanitya M., Lewis C.E., Grunfeld C. (2012). Relationship between MRI-Measured Bone Marrow Adipose Tissue and Hip and Spine Bone Mineral Density in African-American and Caucasian Participants: The CARDIA Study. J. Clin. Endocrinol. Metab..

[84] Yeung D.K., Griffith J.F., Antonio G.E., Lee F.K., Woo J., Leung P.C. (2005). Osteoporosis is associated with increased marrow fat content and decreased marrow fat unsaturation: A proton MR spectroscopy study. J. Magn. Reson. Imaging.

[85] Griffith J.F., Yeung D.K., Antonio G.E., Wong S.Y., Kwok T.C., Woo J., Leung P.C. (2006). Vertebral marrow fat content and diffusion and perfusion indexes in women with varying bone density: MR evaluation. Radiology.

[86] Schellinger D., Lin C.S., Hatipoglu H.G., Fertikh D. (2001). Potential value of vertebral proton MR spectroscopy in determining bone weakness. Am. J. Neuroradiol..

[87] Bandirali M., Di Leo G., Papini G.D.E., Messina C., Sconfienza L.M., Ulivieri F.M., Sardanelli F. (2015). A new diagnostic score to detect osteoporosis in patients undergoing lumbar spine MRI. Eur. Radiol..

[88] Binkley N., Krueger D., Vallarta-Ast N. (2003). An overlying fat panniculus affects femur bone mass measurement. J. Clin. Densitom..

[89] Blake G.M., McKeeney D.B., Chhaya S.C., Ryan P.J., Fogelman I. (1992). Dual energy X-ray absorptiometry: The effects of beam hardening on bone density measurements. Med. Phys..

[90] Weigert J., Cann C. (1999). DXA in obese patients: Are normal values really normal. J. Womens Imaging.

[91] Löffler M.T., Jacob A., Valentinitsch A., Rienmüller A., Zimmer C., Ryang Y.M., Baum T., Kirschke J.S. (2019). Improved prediction of incident vertebral fractures using opportunistic QCT compared to DXA. Eur. Radiol..

[92] Engelke K., Lang T., Khosla S., Qin L., Zysset P., Leslie W.D., Shepherd J.A., Schousboe J.T. (2015). Clinical Use of Quantitative Computed Tomography (QCT) of the Hip in the Management of Osteoporosis in Adults: The 2015 ISCD Official Positions-Part I. J. Clin. Densitom..

[93] Cann C.E., Adams J.E., Brown J.K., Brett A.D. (2014). CTXA hip—An extension of classical DXA measurements using quantitative CT. PLoS ONE.

[94] American College of Radiology (2008). ACR–SPR–SSR Practice Parameter for the Perfomance Of Musculoskeletal Quantitative Computed Tomography (QCT). https://www.acr.org/-/media/ACR/Files/Practice-Parameters/qct.pdf/.

[95] Lichtenstein A., Ferreira-Júnior M., Sales M.M., Aguiar F.B., Fonseca L.A., Sumita N.M., Duarte A.J. (2013). Vitamin D: Non-skeletal actions and rational use. Rev. Assoc. Med. Bras..

[96] Holick M.F. (2004). Sunlight and vitamin D for bone health and prevention of autoimmune diseases, cancers, and cardiovascular disease. Am. J. Clin. Nutr..

[97] Panda D.K., Miao D., Bolivar I., Li J., Huo R., Hendy G.N., Goltzman D. (2004). Inactivation of the 25-hydroxyvitamin D 1alpha-hydroxylase and vitamin D receptor demonstrates independent and interdependent effects of calcium and vitamin D on skeletal and mineral homeostasis. J. Biol. Chem..

[98] Dusso A.S., Thadhani R., Slatopolsky E. (2004). Vitamin D receptor and analogs. Semin Nephrol..

[99] Schmidt T., Ebert K., Rolvien T., Oehler N., Lohmann J., Papavero L., Kothe R., Amling M., Barvencik F., Mussawy H. (2018). A retrospective analysis of bone mineral status in patients requiring spinal surgery. BMC Musculoskelet. Disord..

[100] Kerezoudis P., Rinaldo L., Drazin D., Kallmes D., Krauss W., Hassoon A., Bydon M. (2016). Association Between Vitamin D Deficiency and Outcomes Following Spinal Fusion Surgery: A Systematic Review. World Neurosurg.

[101] Monaco M.D., Vallero F., Monaco R.D., Tappero R., Cavanna A. (2006). 25-Hydroxyvitamin D, parathyroid hormone, and functional recovery after hip fracture in elderly patients. J. Bone Miner. Metab..

[102] Prentice R., Pettinger M., Jackson R., Wactawski-Wende J., Lacroix A., Anderson G., Chlebowski R., Manson J., Van Horn L., Vitolins M. (2013). Health risks and benefits from calcium and vitamin D supplementation: Women’s Health Initiative clinical trial and cohort study. Osteoporos. Int..

[103] Tang B.M., Eslick G.D., Nowson C., Smith C., Bensoussan A. (2007). Use of calcium or calcium in combination with vitamin D supplementation to prevent fractures and bone loss in people aged 50 years and older: A meta-analysis. Lancet.

[104] Mowé M., Haug E., Bøhmer T. (1999). Low serum calcidiol concentration in older adults with reduced muscular function. J. Am. Geriatr. Soc..

[105] Dhesi J.K., Bearne L.M., Moniz C., Hurley M.V., Jackson S.H., Swift C.G., Allain T.J. (2002). Neuromuscular and psychomotor function in elderly subjects who fall and the relationship with vitamin D status. J. Bone Miner. Res..

[106] Pfeifer M., Begerow B., Minne H., Schlotthauer T., Pospeschill M., Scholz M., Lazarescu A., Pollähne W. (2001). Vitamin D status, trunk muscle strength, body sway, falls, and fractures among 237 postmenopausal women with osteoporosis. Exp. Clin. Endocrinol. Diabetes.

[107] Stein M.S., Wark J.D., Scherer S.C., Walton S.L., Chick P., Di Carlantonio M., Zajac J.D., Flicker L. (1999). Falls relate to vitamin D and parathyroid hormone in an Australian nursing home and hostel. J. Am. Geriatr. Soc..

[108] Isaia G., Giorgino R., Rini G., Bevilacqua M., Maugeri D., Adami S. (2003). Prevalence of hypovitaminosis D in elderly women in Italy: Clinical consequences and risk factors. Osteoporos. Int..

[109] Bischoff H.A., Stahelin H.B., Urscheler N., Ehrsam R., Vonthein R., Perrig-Chiello P., Tyndall A., Theiler R. (1999). Muscle strength in the elderly: Its relation to vitamin D metabolites. Arch. Phys. Med. Rehabil..

[110] Ravindra V.M., Godzik J., Dailey A.T., Schmidt M.H., Bisson E.F., Hood R.S., Cutler A., Ray W.Z. (2015). Vitamin D Levels and 1-Year Fusion Outcomes in Elective Spine Surgery: A Prospective Observational Study. Spine.

[111] Formby P.M., Kang D.G., Helgeson M.D., Wagner S.C. (2016). Clinical and Radiographic Outcomes of Transforaminal Lumbar Interbody Fusion in Patients with Osteoporosis. Global Spine J..

[112] Wong R.M.Y., Wong P.Y., Liu C., Wong H.W., Chung Y.L., Chow S.K.H., Law S.W., Cheung W.H. (2022). The imminent risk of a fracture-existing worldwide data: A systematic review and meta-analysis. Osteoporos. Int..

[113] Johansson H., Siggeirsdottir K., Harvey N.C., Oden A., Gudnason V., McCloskey E., Sigurdsson G., Kanis J.A. (2017). Imminent risk of fracture after fracture. Osteoporos. Int..

[114] Wu C.H., Tu S.T., Chang Y.F., Chan D.C., Chien J.T., Lin C.H., Singh S., Dasari M., Chen J.F., Tsai K.S. (2018). Fracture liaison services improve outcomes of patients with osteoporosis-related fractures: A systematic literature review and meta-analysis. Bone.

[115] Genant H.K., Engelke K., Bolognese M.A., Mautalen C., Brown J.P., Recknor C., Goemaere S., Fuerst T., Yang Y.C., Grauer A. (2017). Effects of Romosozumab Compared With Teriparatide on Bone Density and Mass at the Spine and Hip in Postmenopausal Women With Low Bone Mass. J. Bone Miner. Res..

[116] Cosman F., Crittenden D.B., Ferrari S., Khan A., Lane N.E., Lippuner K., Matsumoto T., Milmont C.E., Libanati C., Grauer A. (2018). FRAME Study: The Foundation Effect of Building Bone With 1 Year of Romosozumab Leads to Continued Lower Fracture Risk After Transition to Denosumab. J. Bone Miner. Res..

[117] Brown J.P., Engelke K., Keaveny T.M., Chines A., Chapurlat R., Foldes A.J., Nogues X., Civitelli R., De Villiers T., Massari F. (2021). Romosozumab improves lumbar spine bone mass and bone strength parameters relative to alendronate in postmenopausal women: Results from the Active-Controlled Fracture Study in Postmenopausal Women With Osteoporosis at High Risk (ARCH) trial. J. Bone Miner. Res..

[118] McClung M.R., Bolognese M.A., Brown J.P., Reginster J.Y., Langdahl B.L., Shi Y., Timoshanko J., Libanati C., Chines A., Oates M.K. (2021). Skeletal responses to romosozumab after 12 months of denosumab. JBMR Plus.

[119] Huang S., Lin H., Zhu X., Chen X., Fan L., Liu C. (2014). Zoledronic acid increases bone mineral density and improves health-related quality of life over two years of treatment in Chinese women with postmenopausal osteoporosis. Endokrynol. Pol..

[120] Augoulea A., Tsakonas E., Triantafyllopoulos I., Rizos D., Armeni E., Tsoltos N., Tournis S., Deligeoroglou E., Antoniou A., Lambrinoudaki I. (2017). Comparative effects of denosumab or bisphosphonate treatment on bone mineral density and calcium metabolism in postmenopausal women. J. Musculoskelet. Neuronal. Interact..

[121] Kendler D., Chines A., Clark P., Ebeling P.R., McClung M., Rhee Y., Huang S., Stad R.K. (2020). Bone mineral density after transitioning from denosumab to alendronate. J. Clin. Endocrinol. Metab..

[122] Miller P.D., Hattersley G., Riis B.J., Williams G.C., Lau E., Russo L.A., Alexandersen P., Zerbini C.A.F., Hu M.Y., Harris A.G. (2016). Effect of Abaloparatide vs. Placebo on New Vertebral Fractures in Postmenopausal Women With Osteoporosis: A Randomized Clinical Trial. JAMA.

[123] McClung M.R., Harvey N.C., Fitzpatrick L.A., Miller P.D., Hattersley G., Wang Y., Cosman F. (2018). Effects of abaloparatide on bone mineral density and risk of fracture in postmenopausal women aged 80 years or older with osteoporosis. Menopause.

[124] Saag K.G., Williams S.A., Wang Y., Weiss R.J., Cauley J.A. (2020). Effect of Abaloparatide on Bone Mineral Density and Fracture Incidence in a Subset of Younger Postmenopausal Women with Osteoporosis at High Risk for Fracture. Clin. Ther..

[125] Eastell R., Nickelsen T., Marin F., Barker C., Hadji P., Farrerons J., Audran M., Boonen S., Brixen K., Gomes J.M. (2009). Sequential treatment of severe postmenopausal osteoporosis after teriparatide: Final results of the randomized, controlled European Study of Forsteo (EUROFORS). J. Bone Miner. Res..

[126] Shane E., Shiau S., Recker R.R., Lappe J.M., Agarwal S., Kamanda-Kosseh M., Bucovsky M., Stubby J., Cohen A. (2021). Denosumab After Teriparatide in Premenopausal Women With Idiopathic Osteoporosis. J. Clin. Endocrinol. Metab..

[127] Cummings S.R., Martin J.S., McClung M.R., Siris E.S., Eastell R., Reid I.R., Delmas P., Zoog H.B., Austin M., Wang A. (2009). Denosumab for Prevention of Fractures in Postmenopausal Women with Osteoporosis. N. Engl. J. Med..

[128] Drake M.T., Clarke B.L., Khosla S. (2008). Bisphosphonates: Mechanism of action and role in clinical practice. Mayo Clin. Proc..

[129] Roelofs A.J., Thompson K., Gordon S., Rogers M.J. (2006). Molecular mechanisms of action of bisphosphonates: Current status. Clin. Cancer Res..

[130] Cremers S., Papapoulos S. (2011). Pharmacology of bisphosphonates. Bone.

[131] Hanley D.A., Adachi J.D., Bell A., Brown V. (2012). Denosumab: Mechanism of action and clinical outcomes. Int. J. Clin. Pract..

[132] Coxon F.P., Thompson K., Roelofs A.J., Ebetino F.H., Rogers M.J. (2008). Visualizing mineral binding and uptake of bisphosphonate by osteoclasts and non-resorbing cells. Bone.

[133] Russell R.G., Watts N.B., Ebetino F.H., Rogers M.J. (2008). Mechanisms of action of bisphosphonates: Similarities and differences and their potential influence on clinical efficacy. Osteoporos. Int..

[134] Murakami H., Takahashi N., Sasaki T., Udagawa N., Tanaka S., Nakamura I., Zhang D., Barbier A., Suda T. (1995). A possible mechanism of the specific action of bisphosphonates on osteoclasts: Tiludronate preferentially affects polarized osteoclasts having ruffled borders. Bone.

[135] Russell R.G. (2011). Bisphosphonates: The first 40 years. Bone.

[136] Cremers S.C., Pillai G., Papapoulos S.E. (2005). Pharmacokinetics/pharmacodynamics of bisphosphonates: Use for optimisation of intermittent therapy for osteoporosis. Clin. Pharmacokinet.

[137] Weiss H.M., Pfaar U., Schweitzer A., Wiegand H., Skerjanec A., Schran H. (2008). Biodistribution and plasma protein binding of zoledronic acid. Drug Metab Dispos.

[138] Carnevale V., Dicembrino F., Frusciante V., Chiodini I., Minisola S., Scillitani A. (2000). Different patterns of global and regional skeletal uptake of 99mTc-methylene diphosphonate with age: Relevance to the pathogenesis of bone loss. J. Nucl. Med..

[139] Israel O., Front D., Hardoff R., Ish-Shalom S., Jerushalmi J., Kolodny G.M. (1991). In vivo SPECT quantitation of bone metabolism in hyperparathyroidism and thyrotoxicosis. J. Nucl. Med..

[140] Boivin G.Y., Chavassieux P.M., Santora A.C., Yates J., Meunier P.J. (2000). Alendronate increases bone strength by increasing the mean degree of mineralization of bone tissue in osteoporotic women. Bone.

[141] Seeman E. (2003). Bone quality. Osteoporos. Int..

[142] Adler R.A., El-Hajj Fuleihan G., Bauer D.C., Camacho P.M., Clarke B.L., Clines G.A., Compston J.E., Drake M.T., Edwards B.J., Favus M.J. (2016). Managing Osteoporosis in Patients on Long-Term Bisphosphonate Treatment: Report of a Task Force of the American Society for Bone and Mineral Research. J. Bone Miner. Res..

[143] Deardorff W.J., Cenzer I., Nguyen B., Lee S.J. (2022). Time to Benefit of Bisphosphonate Therapy for the Prevention of Fractures Among Postmenopausal Women With Osteoporosis: A Meta-analysis of Randomized Clinical Trials. JAMA Intern. Med..

[144] Black D.M., Schwartz A.V., Ensrud K.E., Cauley J.A., Levis S., Quandt S.A., Satterfield S., Wallace R.B., Bauer D.C., Palermo L. (2006). Effects of continuing or stopping alendronate after 5 years of treatment: The Fracture Intervention Trial Long-term Extension (FLEX): A randomized trial. JAMA.

[145] Schwartz A.V., Bauer D.C., Cummings S.R., Cauley J.A., Ensrud K.E., Palermo L., Wallace R.B., Hochberg M.C., Feldstein A.C., Lombardi A. (2010). Efficacy of continued alendronate for fractures in women with and without prevalent vertebral fracture: The FLEX trial. J. Bone Miner. Res..

[146] Mellstrom D.D., Sorensen O.H., Goemaere S., Roux C., Johnson T.D., Chines A.A. (2004). Seven years of treatment with risedronate in women with postmenopausal osteoporosis. Calcif. Tissue Int..

[147] Black D.M., Reid I.R., Boonen S., Bucci-Rechtweg C., Cauley J.A., Cosman F., Cummings S.R., Hue T.F., Lippuner K., Lakatos P. (2012). The effect of 3 versus 6 years of zoledronic acid treatment of osteoporosis: A randomized extension to the HORIZON-Pivotal Fracture Trial (PFT). J. Bone Miner. Res..

[148] Shoback D., Rosen C.J., Black D.M., Cheung A.M., Murad M.H., Eastell R. (2020). Pharmacological Management of Osteoporosis in Postmenopausal Women: An Endocrine Society Guideline Update. J. Clin. Endocrinol. Metab..

[149] Bone H.G., Hosking D., Devogelaer J.P., Tucci J.R., Emkey R.D., Tonino R.P., Rodriguez-Portales J.A., Downs R.W., Gupta J., Santora A.C. (2004). Ten years’ experience with alendronate for osteoporosis in postmenopausal women. N. Engl. J. Med..

[150] Watts N.B., Chines A., Olszynski W.P., McKeever C.D., McClung M.R., Zhou X., Grauer A. (2008). Fracture risk remains reduced one year after discontinuation of risedronate. Osteoporos. Int..

[151] Hayes K., Brown K., Cheung A., Kim S., Juurlink D., Cadarette S.M. (2022). Comparative Fracture Risk During Osteoporosis Drug Holidays After Long-Term Risedronate Versus Alendronate Therapy: A Propensity Score-Matched Cohort Study. Ann. Intern. Med..

[152] Diez-Perez A., Naylor K.E., Abrahamsen B., Agnusdei D., Brandi M.L., Cooper C., Dennison E., Eriksen E.F., Gold D.T., Guañabens N. (2017). International Osteoporosis Foundation and European Calcified Tissue Society Working Group. Recommendations for the screening of adherence to oral bisphosphonates. Osteoporos. Int..

[153] Fretes N., Vellios E., Sharma A., Ajiboye R.M. (2020). Radiographic and functional outcomes of bisphosphonate use in lumbar fusion: A systematic review and meta-analysis of comparative studies. Eur. Spine J..

[154] Tu C.W., Huang K.F., Hsu H.T., Li H.Y., Yang S.S., Chen Y.C. (2014). Zoledronic acid infusion for lumbar interbody fusion in osteoporosis. J. Surg. Res..

[155] Xue D., Li F., Chen G., Yan S., Pan Z. (2014). Do bisphosphonates affect bone healing? A meta-analysis of randomized controlled trials. J. Orthop. Surg. Res..

[156] Edwards B.J., Bunta A.D., Lane J., Odvina C., Rao D.S., Raisch D.W., McKoy J.M., Omar I., Belknap S.M., Garg V. (2013). Bisphosphonates and nonhealing femoral fractures: Analysis of the FDA Adverse Event Reporting System (FAERS) and international safety efforts: A systematic review from the Research on Adverse Drug Events And Reports (RADAR) project. J. Bone Joint Surg. Am..

[157] Cosman F., Nieves J.W., Dempster D.W. (2017). Treatment Sequence Matters: Anabolic and Antiresorptive Therapy for Osteoporosis. J. Bone Miner. Res..

[158] Austin M., Yang Y.C., Vittinghoff E., Adami S., Boonen S., Bauer D.C., Bianchi G., Bolognese M.A., Christiansen C., Eastell R. (2012). Relationship between bone mineral density changes with denosumab treatment and risk reduction for vertebral and nonvertebral fractures. J. Bone Miner. Res..

[159] Tsourdi E., Langdahl B., Cohen-Solal M., Aubry-Rozier B., Eriksen E.F., Guanabens N., Obermayer-Pietsch B., Ralston S.H., Eastell R., Zillikens M.C. (2017). Discontinuation of Denosumab therapy for osteoporosis: A systematic review and position statement by ECTS. Bone.

[160] Anastasilakis A.D., Makras P., Yavropoulou M.P., Tabacco G., Naciu A.M., Palermo A. (2021). Denosumab Discontinuation and the Rebound Phenomenon: A Narrative Review. J. Clin. Med..

[161] Aubry-Rozier B., Liebich G.S.D. Can we avoid the loss of bone mineral density one year after denosumab discontinuation? The Reolaus bone project. Proceedings of the European Congress of Rheumatology, EULAR 2019.

[162] Kendler D.L., Roux C., Benhamou C.L. (2010). Effects of denosumab on bone mineral density and bone turnover in postmenopausal women transitioning from alendronate therapy. J. Bone Miner. Res..

[163] Bone H.G., Wagman R.B., Brandi M.L., Brown J.P., Chapurlat R., Cummings S.R., Czerwinski E., Fahrleitner-Pammer A., Kendler D.L., Lippuner K. (2017). 10 years of denosumab treatment in postmenopausal women with osteoporosis: Results from the phase 3 randomised FREEDOM trial and open-label extension. Lancet Diabetes Endocrinol..

[164] Makras P., Appelman-Dijkstra N.M., Papapoulos S.E., van Wissen S., Winter E.M., Polyzos S.A., Yavropoulou M.P., Anastasilakis A.D. (2021). The Duration of Denosumab Treatment and the Efficacy of Zoledronate to Preserve Bone Mineral Density After Its Discontinuation. J. Clin. Endocrinol. Metab..

[165] McDonald M.M., Khoo W.H., Ng P.Y., Xiao Y., Zamerli J., Thatcher P., Kyaw W., Pathmanandavel K., Grootveld A.K., Moran I. (2021). Osteoclasts recycle via osteomorphs during RANKL-stimulated bone resorption. Cell.

[166] Adami S., Libanati C., Boonen S., Cummings S.R., Ho P.R., Wang A., Siris E., Lane J. (2012). Denosumab treatment in postmenopausal women with osteoporosis does not interfere with fracture-healing: Results from the FREEDOM trial. JBJS.

[167] Rolighed L., Rejnmark L., Christiansen P. (2014). Bone Involvement in Primary Hyperparathyroidism and Changes After Parathyroidectomy. Eur. Endocrinol..

[168] Dobnig H., Sipos A., Jiang Y., Fahrleitner-Pammer A., Ste-Marie L.G., Gallagher J.C., Pavo I., Wang J., Eriksen E.F. (2005). Early changes in biochemical markers of bone formation correlate with improvements in bone structure during teriparatide therapy. J. Clin. Endocrinol. Metab..

[169] Bernhardsson M., Aspenberg P. (2018). Abaloparatide versus teriparatide: A head to head comparison of effects on fracture healing in mouse models. Acta Orthop..

[170] Jiang Y., Zhao J.J., Mitlak B.H., Wang O., Genant H.K., Eriksen E.F. (2003). Recombinant human parathyroid hormone (1–34) [teriparatide] improves both cortical and cancellous bone structure. J. Bone Miner. Res..

[171] Boyce E.G., Mai Y., Pham C. (2018). Abaloparatide: Review of a Next-Generation Parathyroid Hormone Agonist. Ann. Pharmacother..

[172] Choksi P., Jepsen K.J., Clines G.A. (2018). The challenges of diagnosing osteoporosis and the limitations of currently available tools. Clin. Diabetes Endocrinol..

[173] Hattersley G., Dean T., Corbin B.A., Bahar H., Gardella T.J. (2016). Binding selectivity of abaloparatide for PTH-type-1-receptor conformations and effects on downstream signaling. Endocrinology.

[174] Bhattacharyya S., Pal S., Chattopadhyay N. (2019). Abaloparatide, the second generation osteoanabolic drug: Molecular mechanisms underlying its advantages over the first-in-class teriparatide. Biochem. Pharmacol..

[175] Carlson B.C., Robinson W.A., Wanderman N.R., Sebastian A.S., Nassr A., Freedman B.A., Anderson P.A. (2019). A Review and Clinical Perspective of the Impact of Osteoporosis on the Spine. Geriatr. Orthop. Surg. Rehabil..

[176] Ohtori S., Orita S., Yamauchi K., Eguchi Y., Ochiai N., Kuniyoshi K., Aoki Y., Nakamura J., Miyagi M., Suzuki M. (2015). More than 6 Months of Teriparatide Treatment Was More Effective for Bone Union than Shorter Treatment Following Lumbar Posterolateral Fusion Surgery. Asian Spine J..

[177] Miyakoshi N., Aizawa T., Sasaki S., Ando S., Maekawa S., Aonuma H., Tsuchie H., Sasaki H., Kasukawa Y., Shimada Y. (2015). Healing of bisphosphonate-associated atypical femoral fractures in patients with osteoporosis: A comparison between treatment with and without teriparatide. J. Bone Miner. Metab..

[178] Inoue G., Ueno M., Nakazawa T., Imura T., Saito W., Uchida K., Ohtori S., Toyone T., Takahira N., Takaso M. (2014). Teriparatide increases the insertional torque of pedicle screws during fusion surgery in patients with postmenopausal osteoporosis. J. Neurosurg. Spine.

[179] Leder B.Z., O’Dea L.S.L., Zanchetta J.R., Kumar P., Banks K., McKay K., Lyttle C.R., Hattersley G. (2015). Effects of abaloparatide, a human parathyroid hormone-related peptide analog, on bone mineral density in postmenopausal women with osteoporosis. J. Clin. Endocrinol. Metab..

[180] Bilezikian J., Hattersley G., Fitzpatrick L., Harris A., Shevroja E., Banks K., Leder B., Zanchetta J., Hans D. (2018). Abaloparatide-SC improves trabecular microarchitecture as assessed by trabecular bone score (TBS): A 24-week randomized clinical trial. Osteoporos. Int..

[181] Watts N., Hattersley G., Fitzpatrick L., Wang Y., Williams G., Miller P., Cosman F. (2019). Abaloparatide effect on forearm bone mineral density and wrist fracture risk in postmenopausal women with osteoporosis. Osteoporos. Int..

[182] Langdahl B.L., Silverman S., Fujiwara S., Saag K., Napoli N., Soen S., Enomoto H., Melby T.E., Disch D.P., Marin F. (2018). Real-world effectiveness of teriparatide on fracture reduction in patients with osteoporosis and comorbidities or risk factors for fractures: Integrated analysis of 4 prospective observational studies. Bone.

[183] Cosman F., Hattersley G., Hu M.y., Williams G.C., Fitzpatrick L.A., Black D.M. (2017). Effects of Abaloparatide-SC on Fractures and Bone Mineral Density in Subgroups of Postmenopausal Women With Osteoporosis and Varying Baseline Risk Factors. J. Bone Miner. Res..

[184] Miller P.D., Lewiecki E.M., Krohn K., Schwartz E. (2021). Teriparatide: Label changes and identifying patients for long-term use. Cleve Clin. J. Med..

[185] BioSpace (2021). Radius Announces Update on TYMLOS^®^ (Abaloparatide) Label. https://www.biospace.com/article/releases/radius-announces-update-on-tymlos-abaloparatide-label.

[186] Cosman F., Nieves J., Woelfert L., Formica C., Gordon S., Shen V., Lindsay R. (2001). Parathyroid hormone added to established hormone therapy: Effects on vertebral fracture and maintenance of bone mass after parathyroid hormone withdrawal. J. Bone Miner. Res..

[187] Cohen A., Kamanda-Kosseh M., Recker R.R., Lappe J.M., Dempster D.W., Zhou H., Cremers S., Bucovsky M., Stubby J., Shane E. (2015). Bone Density After Teriparatide Discontinuation in Premenopausal Idiopathic Osteoporosis. J. Clin. Endocrinol. Metab..

[188] Shah A.D., Shoback D., Lewiecki E.M. (2015). Sclerostin inhibition: A novel therapeutic approach in the treatment of osteoporosis. Int. J. Womens Health.

[189] Wang W., Wang E.Q., Balthasar J.P. (2008). Monoclonal antibody pharmacokinetics and pharmacodynamics. Clin. Pharmacol. Ther..

[190] Keizer R.J., Huitema A.D., Schellens J.H., Beijnen J.H. (2010). Clinical pharmacokinetics of therapeutic monoclonal antibodies. Clin. Pharmacokinet..

[191] Lim S.Y., Bolster M.B. (2017). Profile of romosozumab and its potential in the management of osteoporosis. Drug Des. Devel. Ther..

[192] Chavassieux P., Chapurlat R., Portero-Muzy N., Roux J.P., Garcia P., Brown J.P., Libanati C., Boyce R.W., Wang A., Grauer A. (2019). Bone-Forming and Antiresorptive Effects of Romosozumab in Postmenopausal Women With Osteoporosis: Bone Histomorphometry and Microcomputed Tomography Analysis After 2 and 12 Months of Treatment. J. Bone Miner. Res..

[193] Saag K.G., Petersen J., Brandi M.L., Karaplis A.C., Lorentzon M., Thomas T., Maddox J., Fan M., Meisner P.D., Grauer A. (2017). Romosozumab or Alendronate for Fracture Prevention in Women with Osteoporosis. N. Engl. J. Med..

[194] Cosman F., Crittenden D.B., Adachi J.D., Binkley N., Czerwinski E., Ferrari S., Hofbauer L.C., Lau E., Lewiecki E.M., Miyauchi A. (2016). Romosozumab Treatment in Postmenopausal Women with Osteoporosis. N. Engl. J. Med..

[195] Cosman F., Crittenden D.B., Ferrari S., Lewiecki E.M., Jaller-Raad J., Zerbini C., Milmont C.E., Meisner P.D., Libanati C., Grauer A. (2018). Romosozumab FRAME study: A post hoc analysis of the role of regional background fracture risk on nonvertebral fracture outcome. J. Bone Miner. Res..

[196] Tian A., Jia H., Zhu S., Lu B., Li Y., Ma J., Ma X. (2021). Romosozumab versus Teriparatide for the Treatment of Postmenopausal Osteoporosis: A Systematic Review and Meta-analysis through a Grade Analysis of Evidence. Orthop. Surg..

[197] Schemitsch E.H., Miclau T., Karachalios T., Nowak L.L., Sancheti P., Poolman R.W., Caminis J., Daizadeh N., Dent-Acosta R.E., Egbuna O. (2020). A Randomized, Placebo-Controlled Study of Romosozumab for the Treatment of Hip Fractures. J. Bone Joint Surg. Am..

[198] Yoshiki F., Nishikawa A., Taketsuna M., Kajimoto K., Enomoto H. (2017). Efficacy and safety of teriparatide in bisphosphonate-pretreated and treatment-naive patients with osteoporosis at high risk of fracture: Post hoc analysis of a prospective observational study. J. Orthop. Sci..

[199] Langdahl B.L., Libanati C., Crittenden D.B., Bolognese M.A., Brown J.P., Daizadeh N.S., Dokoupilova E., Engelke K., Finkelstein J.S., Genant H.K. (2017). Romosozumab (sclerostin monoclonal antibody) versus teriparatide in postmenopausal women with osteoporosis transitioning from oral bisphosphonate therapy: A randomised, open-label, phase 3 trial. Lancet.

[200] Leder B.Z., Tsai J.N., Uihlein A.V., Wallace P.M., Lee H., Neer R.M., Burnett-Bowie S.A. (2015). Denosumab and teriparatide transitions in postmenopausal osteoporosis (the DATA-Switch study): Extension of a randomised controlled trial. Lancet.

[201] Kendler D., Bone H., Massari F., Gielen E., Palacios S., Maddox J., Yan C., Yue S., Dinavahi R., Libanati C. (2019). Bone mineral density gains with a second 12-month course of romosozumab therapy following placebo or denosumab. Osteoporos. Int..

[202] Anastasilakis A.D., Papapoulos S.E., Polyzos S.A., Appelman-Dijkstra N.M., Makras P. (2019). Zoledronate for the Prevention of Bone Loss in Women Discontinuing Denosumab Treatment. A Prospective 2-Year Clinical Trial. J. Bone Miner. Res..

[203] Lewiecki E.M., Dinavahi R.V., Lazaretti-Castro M., Ebeling P.R., Adachi J.D., Miyauchi A., Gielen E., Milmont C.E., Libanati C., Grauer A. (2019). One Year of Romosozumab Followed by Two Years of Denosumab Maintains Fracture Risk Reductions: Results of the FRAME Extension Study. J. Bone Miner. Res..

[204] Niimi R., Kono T., Nishihara A., Hasegawa M., Kono T., Sudo A. (2018). Efficacy of Switching From Teriparatide to Bisphosphonate or Denosumab: A Prospective, Randomized, Open-Label Trial. JBMR Plus.

[205] Markiewicz M.R., Margarone J.E.R., Campbell J.H., Aguirre A. (2005). Bisphosphonate-associated osteonecrosis of the jaws: A review of current knowledge. J. Am. Dent. Assoc..

[206] Bagan J.V., Jimenez Y., Murillo J., Hernandez S., Poveda R., Sanchis J.M., Diaz J.M., Scully C. (2006). Jaw osteonecrosis associated with bisphosphonates: Multiple exposed areas and its relationship to teeth extractions. Study of 20 cases. Oral Oncol..

[207] Lo J.C., O’Ryan F.S., Gordon N.P., Yang J., Hui R.L., Martin D., Hutchinson M., Lathon P.V., Sanchez G., Silver P. (2010). Prevalence of osteonecrosis of the jaw in patients with oral bisphosphonate exposure. J. Oral Maxillofac. Surg..

[208] Boquete-Castro A., Gomez-Moreno G., Calvo-Guirado J.L., Aguilar-Salvatierra A., Delgado-Ruiz R.A. (2016). Denosumab and osteonecrosis of the jaw. A systematic analysis of events reported in clinical trials. Clin. Oral Implants Res..

[209] Shibahara T. (2019). Antiresorptive Agent-Related Osteonecrosis of the Jaw (ARONJ): A Twist of Fate in the Bone. Tohoku J. Exp. Med..

[210] Ruggiero S.L., Mehrotra B., Rosenberg T.J., Engroff S.L. (2004). Osteonecrosis of the jaws associated with the use of bisphosphonates: A review of 63 cases. J. Oral Maxillofac. Surg..

[211] Grbic J.T., Black D.M., Lyles K.W., Reid D.M., Orwoll E., McClung M., Bucci-Rechtweg C., Su G. (2010). The incidence of osteonecrosis of the jaw in patients receiving 5 milligrams of zoledronic acid: Data from the health outcomes and reduced incidence with zoledronic acid once yearly clinical trials program. J. Am. Dent. Assoc..

[212] Everts-Graber J., Lehmann D., Burkard J.P., Schaller B., Gahl B., Hauselmann H., Studer U., Ziswiler H.R., Reichenbach S., Lehmann T. (2022). Risk of Osteonecrosis of the Jaw Under Denosumab Compared to Bisphosphonates in Patients With Osteoporosis. J. Bone Miner. Res..

[213] Sim I.W., Borromeo G.L., Tsao C., Hardiman R., Hofman M.S., Papatziamos Hjelle C., Siddique M., Cook G.J.R., Seymour J.F., Ebeling P.R. (2020). Teriparatide Promotes Bone Healing in Medication-Related Osteonecrosis of the Jaw: A Placebo-Controlled, Randomized Trial. J. Clin. Oncol..

[214] Black D.M., Geiger E.J., Eastell R., Vittinghoff E., Li B.H., Ryan D.S., Dell R.M., Adams A.L. (2020). Atypical femur fracture risk versus fragility fracture prevention with bisphosphonates. N. Engl. J. Med..

[215] Schilcher J., Aspenberg P. (2009). Incidence of stress fractures of the femoral shaft in women treated with bisphosphonate. Acta Orthop..

[216] Bauer D.C. (2012). Atypical Femoral Fracture Risk in Patients Treated With Bisphosphonates: Comment on “Increasing Occurrence of Atypical Femoral Fractures Associated With Bisphosphonate Use”. Arch. Intern. Med..

[217] Dell R.M., Adams A.L., Greene D.F., Funahashi T.T., Silverman S.L., Eisemon E.O., Zhou H., Burchette R.J., Ott S.M. (2012). Incidence of atypical nontraumatic diaphyseal fractures of the femur. J. Bone Miner. Res..

[218] Shane E., Burr D., Abrahamsen B., Adler R.A., Brown T.D., Cheung A.M., Cosman F., Curtis J.R., Dell R., Dempster D.W. (2014). Atypical subtrochanteric and diaphyseal femoral fractures: Second report of a task force of the American Society for Bone and Mineral Research. J. Bone Miner. Res..

[219] Schilcher J., Sandberg O., Isaksson H., Aspenberg P. (2014). Histology of 8 atypical femoral fractures: Remodeling but no healing. Acta Orthop..

[220] Russell R.G.G., Xia Z., Dunford J.E., Oppermann U., Kwaasi A., Hulley P.A., Kavanagh K.L., Triffitt J.T., Lundy M.W., Phipps R.J. (2007). Bisphosphonates: An update on mechanisms of action and how these relate to clinical efficacy. Ann. N. Y. Acad. Sci..

[221] Boskey A., Spevak L., Weinstein R. (2009). Spectroscopic markers of bone quality in alendronate-treated postmenopausal women. Osteoporos. Int..

[222] Demirtas A., Rajapakse C.S., Ural A. (2020). Assessment of the multifactorial causes of atypical femoral fractures using a novel multiscale finite element approach. Bone.

[223] Donnelly E., Saleh A., Unnanuntana A., Lane J.M. (2012). Atypical femoral fractures: Epidemiology, etiology, and patient management. Curr. Opin. Support. Palliat. Care.

[224] Oh Y., Yamamoto K., Hashimoto J., Fujita K., Yoshii T., Fukushima K., Kurosa Y., Wakabayashi Y., Kitagawa M., Okawa A. (2020). Biological activity is not suppressed in mid-shaft stress fracture of the bowed femoral shaft unlike in “typical” atypical subtrochanteric femoral fracture: A proposed theory of atypical femoral fracture subtypes. Bone.

[225] Hirano F., Okuma K.F., Zenke Y., Menuki K., Ohnishi H., Fukuda F., Sakai A., Yamamoto N., Shimakura T., Sano H. (2021). Disturbance of osteonal bone remodeling and high tensile stresses on the lateral cortex in atypical femoral fracture after long-term treatment with Risedronate and Alfacalcidol for osteoporosis. Bone Rep..

[226] Weinstein R.S., Roberson P.K., Manolagas S.C. (2009). Giant osteoclast formation and long-term oral bisphosphonate therapy. N. Engl. J. Med..

[227] Jensen P.R., Andersen T.L., Chavassieux P., Roux J.P., Delaisse J.M. (2021). Bisphosphonates impair the onset of bone formation at remodeling sites. Bone.

[228] Giusti A., Hamdy N.A., Papapoulos S.E. (2010). Atypical fractures of the femur and bisphosphonate therapy: A systematic review of case/case series studies. Bone.

[229] Shane E., Burr D., Ebeling P.R., Abrahamsen B., Adler R.A., Brown T.D., Cheung A.M., Cosman F., Curtis J.R., Dell R. (2010). Atypical subtrochanteric and diaphyseal femoral fractures: Report of a task force of the American Society for Bone and Mineral Research. J. Bone Miner. Res..

[230] Van de Laarschot D.M., McKenna M.J., Abrahamsen B., Langdahl B., Cohen-Solal M., Guañabens N., Eastell R., Ralston S.H., Zillikens M.C. (2020). Medical management of patients after atypical femur fractures: A systematic review and recommendations from the European Calcified Tissue Society. J. Clin. Endocrinol. Metab..

